# Near Equilibrium Calculus of Stem Cells in Application to the Airway Epithelium Lineage

**DOI:** 10.1371/journal.pcbi.1004990

**Published:** 2016-07-18

**Authors:** Zheng Sun, Maksim V. Plikus, Natalia L. Komarova

**Affiliations:** 1 Department of Mathematics, University of California, Irvine, Irvine, California, United States of America; 2 Department of Developmental and Cell Biology, Sue and Bill Gross Stem Cell Research Center and Center for Complex Biological Systems, University of California, Irvine, Irvine, California, United States of America; 3 Department of Ecology and Evolutionary Biology, University of California, Irvine, Irvine, California, United States of America; Complexo Interdisciplinar da Universidade de Lisboa, PORTUGAL

## Abstract

Homeostatic maintenance of tissues is orchestrated by well tuned networks of cellular signaling. Such networks regulate, in a stochastic manner, fates of all cells within the respective lineages. Processes such as symmetric and asymmetric divisions, differentiation, de-differentiation, and death have to be controlled in a dynamic fashion, such that the cell population is maintained at a stable equilibrium, has a sufficiently low level of stochastic variation, and is capable of responding efficiently to external damage. Cellular lineages in real tissues may consist of a number of different cell types, connected by hierarchical relationships, albeit not necessarily linear, and engaged in a number of different processes. Here we develop a general mathematical methodology for near equilibrium studies of arbitrarily complex hierarchical cell populations, under regulation by a control network. This methodology allows us to (1) determine stability properties of the network, (2) calculate the stochastic variance, and (3) predict how different control mechanisms affect stability and robustness of the system. We demonstrate the versatility of this tool by using the example of the airway epithelium lineage. Recent research shows that airway epithelium stem cells divide mostly asymmetrically, while the so-called secretory cells divide predominantly symmetrically. It further provides quantitative data on the recovery dynamics of the airway epithelium, which can include secretory cell de-differentiation. Using our new methodology, we demonstrate that while a number of regulatory networks can be compatible with the observed recovery behavior, the observed division patterns of cells are the most optimal from the viewpoint of homeostatic lineage stability and minimizing the variation of the cell population size. This not only explains the observed yet poorly understood features of airway tissue architecture, but also helps to deduce the information on the still largely hypothetical regulatory mechanisms governing tissue turnover, and lends insight into how different control loops influence the stability and variance properties of cell populations.

## Author Summary

Tissue stability is the basic property of healthy organs, and yet the mechanisms governing the stable, long-term maintenance of cell numbers in tissues are poorly understood. While more and more signaling pathways are being discovered, for the most part it remains unknown how they are being put together by different cell types into complex, nonlinear, hierarchical control networks that, on the one hand, reliably maintain constant cell numbers, and on the other hand, quickly adjust to oversee the robust response to tissue damage. Theoretical approaches can fill the gap by being able to reconstruct the underlying control network, based on the observations about the aspects of cellular dynamics. We argue that while many hypothetical networks may be capable of basic cell lineage maintenance, some are much more efficient from the viewpoint of variance minimization. Thus, we developed a new methodology that can test various control networks for stability, variance, and robustness. In the example of the airway epithelium that we highlight, it turns out that the evolutionary selected, actual architecture coincides with the mathematically optimal solution that minimizes the fluctuations of cell numbers at homeostasis.

## Introduction

All tissues and organs in our bodies can be deconstructed and arranged into phylogenetic cellular lineages. At the base of every lineage lie stem cells (SCs), the long lasting, self-renewing and generally non-differentiated cell type. Progeny of SCs progressively reduce their proliferative potential and concomitantly acquire specialized differentiated characteristics and novel functions. Typically, fully differentiated cells are post-mitotic and have limited life span, and thus require to be constantly replenished from the SC compartment. Proper steady-state maintenance of the lineages, as well as their rapid responses to cellular loss or excessive expansion require checks and balances at all steps of lineage progression, from stem to terminally differentiated cells. Significant advances in our understanding of the SC biology, as well as high potential for SC modulation as a therapeutic solution to a broad range of regenerative disorders, from non-healing wounds to rapid tumor growth [1–4], have inspired a lot of theoretical work in the field of lineage regulation.

The focus of the present study is understanding control networks involved in the homeostasis of healthy tissues. For a given, two- or multi-compartment lineage system, the control of cellular decisions, such as division and death timing, or division type, can be mediated by feedback loops that depend on the current state of cellular population(s), more precisely, on the relative numbers of distinct cell types within the lineage. For example, the decision for a SC to proliferate can depend on whether there is a deficiency either in the SC compartment, or in other downstream compartment(s). Similarly, the decision for a non-SC progenitor to terminally differentiate could depend on the current number of other terminally differentiated cells. In addition to proliferation and differentiation, other cellular events include asymmetric cell divisions, de-differentiation, and apoptosis. Cell numbers change as the result of divisions and deaths. How can the cell lineage system as a whole be regulated to remain at a near-equilibrium? Several cell populations can participate in signaling, and control loops can be both positive and negative, to regulate, in a self-correcting way, the rate at which all of these processes take place. Given a complex system of this kind, we need to be able to evaluate whether the control network is capable of producing stable homeostasis, quantify the magnitude of variances resulting from perturbations, and assess the robustness of the stochastic lineage turnover.

In [5] we considered stochastic dynamics of cellular lineages in a two-compartment model, which included SCs and one type of differentiated cells. We assumed that in such prototypical lineage only three cellular events took place: (i) death of a differentiated cell, (ii) proliferation or, alternatively, (iii) differentiation of a SC. While valuable, this approach has limitations because it only allows two cell types and three processes in the system. More recently, we showed that such two-compartment model can be sufficient to faithfully describe and predict cellular behaviors in relatively simple lineages, such as mammalian epidermis [6]. Considering the value of this methodology, it is important to generalize it and make it applicable for studying a larger class of more complex cellular lineages.

Examples of complex lineages are numerous. Commonly, there are multiple intermediate proliferating cell types, sometimes referred to as transit amplifying cells, between SCs and terminally differentiated post-mitotic cells. Such intermediate progenitors are prominent in the hematopoietic [7, 8], intestinal epithelium [9, 10] and hair follicle epithelium lineages [11–16]. For example, in the hematopoietic lineage, bona fide hematopoietic SCs give rise to common lymphoid and common myeloid progenitors. The latter, in turn, produce granulocyte-macrophage and megakaryocyte–erythroid progenitors [7, 8]. Moreover, lineages often contain more than one type of SCs and more then one distinct type of differentiated cells. For example, there are two principal types of epithelial SCs in the intestinal epithelium, rapidly proliferating crypt base columnar SCs and quiescent +4 SCs [9, 10, 17]. There are also seven distinct differentiated cell types that these SCs can produce: absorptive enterocytes, enteroendocrine cells, Tuft cells, Goblet cells, Paneth cells, M-cells and cup cells [9, 18]. In lineages with more than one SC type, there is often SC-to-SC interchangeability. For instance, crypt base columnar SCs and quiescent +4 SCs in the intestine can interconvert, depending on the conditions—crypt base columnar SCs are sensitive to damage, become largely depleted after irradiation and then restore from radiation-resistant +4 SCs [19–21]. In addition, following depletion, cells can be replenished from the non-SC progenitors via the so called de-differentiation or reprogramming mechanisms. Such is biliary epithelial cells regeneration via hepatocytes reprogramming in the liver following toxin-induced depletion [22, 23]. In the lung, alveolar type-2 cells can reprogram into type-1 cells when the latter are selectively ablated by the hyperoxic injury [24]. Similarly, in the stomach, differentiated secretory Troy+ chief cells can de-differentiate into SCs following genetic depletion of the SC compartment [25]. Ideally, a mathematical framework is needed that is not restricted by a small number of cell types, and can handle this biological variety.

In this paper we present a theoretical framework that allows to study stability, fluctuations, and robustness of near equilibrium cell dynamics for multi-process, multi-compartment lineages. We obtain analytically, in a general case, (i) constraints on the equilibrium rates of all the processes compatible with the existence of a steady state; (ii) the stability conditions for the steady state, and (iii) solutions for the second moments for all the cell populations. The latter describe comprehensively how different components of the control network affect fluctuations of different cell populations. This versatile mathematical framework, which we call “near equilibrium calculus of stem cells”, allows one to perform computations for arbitrarily complex cell lineages, under any regulatory control network. With this new tool, one can attempt several conceptual types of inquiries. One is explanatory: given an observed pattern (for example, the symmetry of cell divisions, or the type and direction of control loops observed), one can attempt to explain why any particular kind of tissue architecture and cell population management logic have evolved, or, more precisely, evaluate if the given control network and the resulting division patterns are in any sense optimal in the context of stability of the system and robustness of its homeostatic maintenance. The second type of application is predictive: if a network regulating a certain system is unknown (or not completely understood), one can hypothesize what type of a network would be compatible with the given observables and at the same time optimal from the viewpoint of robust homeostatic maintenance. Finally, given a regulatory network, one can evaluate the importance of its different components and the influence they exert on the amount of variance experienced by the cell population.

To illustrate the versatility of the method, we apply it to the studies of the airway epithelium system. This system has recently attracted a lot of attention because (1) its key cell types, including stem cells, are well defined, (2) it has tractable two-dimensional organization, and (3) multiple genetic tools have become available to target each of the lineage’s cell types, either to induce cell depletion or gene deletion/mis-expression. In particular, airway epithelium lineage has proven to be a great model system for tracking responses to cell depletion in a semi-quantifiable way—following genetic depletion of a given cellular type, the response of the remaining cells can be precisely measured and tracked in time. Semi-quantitative nature of these recently published experiments provides a plethora of valuable numerical information, which can be modeled.

By using our methodology, we were able to incorporate the available data and come up with a set of control networks that are compatible with the observed recovery patterns of the airway epithelium [26–28]. Further, we concerned ourselves with the general question of tissue design. It has been reported in the recent literature [29] that in the three-compartment cellular lineage of the airway epithelium, the SCs are characterized by mostly asymmetric divisions, while the secretory cells (SecrCs), the next cell type in the differentiation hierarchy, are characterized by mostly symmetric divisions. By using the mathematical approach developed here, we show that (1) predominantly symmetric divisions of the SecrCs is a necessary feature that makes the lineage system compatible with the reportedly slow dynamics of the most differentiated ciliated cells (CilCs), and that (2) predominantly asymmetric divisions of the SCs may be the consequence of the mathematical fact that asymmetric SC divisions, under the other existing constraints of the airway epithelial system, minimize the fluctuations of both SC and SecrC populations.

Our work contributes to the growing computational literature on SC dynamics. Many aspects of SC dynamics have been modeled and studied mathematically. Methodologically, both discrete and continuous computational models have been used, particularly in the context of SC mutagenesis and carcinogenesis [30–41]. In addition to cancer, normal SC behaviors, such as (i) symmetry vs. asymmetry of SC divisions, (ii) SC quiescence vs. proliferative activation, and (iii) progressive lineage specification have been modeled, such as in the hematopoietic system [42–46]. Here, again, both deterministic and stochastic models have been introduced and studied (see the review in [47]). Lineage decision-making controls have been studied deterministically both in the context of minimalistic two-compartment, as well as multi-compartment models [48–52]. Stochastic lineage systems have been considered as well in [53–59], and feedback regulation of SC dynamics has been modeled in [48, 48, 60]. The present approach attempts to generalize the description of SC dynamics in the context of healthy tissue turnover. We strive to create a framework general enough to describe any feasible control network for any hierarchical organization, but at the same time to find a way for analytical understanding of the resulting dynamics, focusing on the role of various control loops in homeostatic maintenance.

## Methods

Suppose there are *n* compartments in a cellular lineage. Cells in different compartments differ by their properties (such as their degree of differentiation, function, etc). The numbers of cells in each compartment are denoted as *i*^1^, …, *i*^*n*^. We further assume the existence of *K* different cellular processes that change the number and/or type of cells in different compartment. Examples of such processes are symmetric proliferations of SCs, death of differentiated cells, or de-differentiaion of intermediate cells.

Let us denote by *Q*_*k*_(*i*^1^, …, *i*^*n*^) the rates at which these processes take place. Here we assume that in principle, these rates can be functions of all the cell populations in the lineage. In reality, not all populations can control each process. Therefore, it is useful to consider partial derivatives of the rates with respect to different population sizes. For example, quantity

$$\frac{\partial Q_{p}}{\partial i^{q}},$$ (1)

where the derivative is evaluated at the equilibrium (the homeostatic state), informs us whether or not process *Q*_*p*_ is regulated by cells in compartment *q*. If the derivative above is positive (negative), then the control is positive (negative). A zero derivative means the absence of control. We sometimes refer to quantities Eq (1) as simply “controls”.

A convenient way to think about all possible controls is in terms of networks, where one set of nodes corresponds to all the compartments and the second set of nodes to all the processes. The existence of a signed edge between a compartment and a process corresponds to the existence of the corresponding control. The magnitude of controls Eq (1) can be presented as weights of the corresponding edges. A stable control network possesses a set of weights that lead to a stable homeostatic state. A minimal network contains the smallest possible number of edges.

Associated with each process, *k*, we further define a vector of associated increments of all the cell populations, (Δ_*k*_ *i*^1^, …, Δ_*k*_ *i*^*n*^). For example, in a three-compartment system consisting of SCs, intermediate cells, and differentiated cells, symmetric proliferation of SCs results in increment (1, 0, 0), death of differentiated cells in increment (0, 0, −1), and de-differentiation of intermediate cells in increment (1, −1, 0). These vectors can be thought of as signatures of all the processes that happen in the lineage.

### Constraints on the equilibrium rates

The equilibrium is defined by *n* algebraic equations for the *n* variables, $\left(i_{*}^{1},\ldots ,i_{*}^{n}\right)$, which are the equilibrium population sizes of all the compartments:

$$\sum_{k=1}^{K}Q_{k}\left(i_{*}^{1},\ldots ,i_{*}^{n}\right)\Delta_{k}i^{1}=0,\ldots ,\sum_{k=1}^{K}Q_{k}\left(i_{*}^{1},\ldots ,i_{*}^{n}\right)\Delta_{k}i^{n}=0.$$ (2)

If the functional form of all the rates *Q*_*k*_ is known, then the equilibria can be determined. In reality, the equilibrium population values can be measured, but the functional form of *Q*_*k*_ is unknown. Therefore, it is more useful to interpret eq (2) as a linear system of equations for the equilibrium rates, which imposes *K* − *n* constraints on the rate values. In other words, only *K* − *n* out of *K* rates can be assigned an independent value at the equilibrium.

### Stability and robustness

Controls of the different processes combine with the cellular increments to form the Jacobian corresponding to the equilibrium point,

$$J=\left\{a_{mj}\right\},\;a_{mj}=\sum_{k=1}^{K}\frac{\partial Q_{k}}{\partial i^{j}}\Delta_{k}i^{m},\;1\leq m,j\leq n,$$ (3)

where the derivatives are assumed to be taken at the equilibrium. It is demonstrated in S1 Text, Section 1, that the eigenvalues of *J* inform us not only of the stability of the deterministic equations, but also of the stability of the system involving higher moments. The control loops define how sparse matrix *J* is.

System robustness can be investigated alongside with stability in the following way. Suppose that the control loops are fixed in the sense that we know the topology of the control network (which cell population controls which process(es)), and the sign of controls. Let us vary the values of nonzero derivatives Eq (1) within some bounds. What portion of the set of parameters corresponds to a stable system? In the most robust scenario, we have a sign-stable matrix *J*, that is, it is stable for all parameter values of the given signs. In a less robust scenario, only a small portion of parameter space corresponds to stability.

### Variance

While the equilibrium constraints and the Jacobian are obtained by deterministic methods, the next step of the analysis is stochastic. Here we extend the methodology developed in [5] and [61] to describe multi-compartment, multi-process systems. Let us denote by *y*_*pq*_ the covariance of the populations in compartments *p* and *q*. It is convenient to form the variance vector,

$$\vec{y}={\left(y_{11},y_{12},\ldots ,y_{nn}\right)}^{T}.$$

Quantities *y*_11_, …, *y*_*nn*_ correspond to second central moments, or the variances, of the cell populations. The covariances and the variances can be determined analytically from (i) the equilibrium rates, (ii) the control values, and (iii) the increments associated with the processes. It is convenient to define matrix *W* as the Kronecker sum

W=J⊕J≡J⊗I+I⊗J, (4)

where matrix *I* represents the identity matrix (see S1 Text, Section 1 for details). This matrix contains the information about the controls at the equilibrium. The information about the equilibrium rates and the increments is combined in an *n*^2^ × 1 vector $\vec{s}={\left(s_{11},s_{12},\ldots ,s_{nn}\right)}^{T}$, which has elements

$$s_{pq}=\sum_{k=1}^{K}Q_{k*}\Delta_{k}i^{p}\Delta_{k}i^{q},\;p,q=1,2,\ldots ,n.$$ (5)

The covariances are then given as solutions of the linear system

$$W\vec{y}=-\vec{s}.$$ (6)

Because of the special form of the matrix *W*, eq (6) is equivalent to the continuous Lyapunov equation,

$$JY+YJ^{T}=-S.$$

Here, *Y* is an *n* × *n* matrix with elements *y*_*pq*_, *J* is the Jacobian (eq (3)), and *S* is an *n* × *n* matrix with elements *s*_*pq*_ (eq (5)). This equation arises in the Lyapunov stability theory and several applications of control theory [62, 63]. Its unique solution *Y* can be expressed in terms of matrix *J* in the following way,

$$Y=\int_{0}^{\infty }\exp\left(Jt\right)S\exp\left(J^{T}t\right)\;dt,$$ (7)

see e.g. [64]. This integral converges as long as all the eigenvalues of the matrix *J* have negative real parts. The diagonal elements of the matrix *Y* give the variance of the cell population numbers.

### Stochastic and deterministic analysis

Both types of analysis (the regular stability analysis and analysis of variance) share some important features, which is expected. For example, if the real parts of all the eigenvalues become larger (and negative) in a certain direction of the parameter space, the variances of all the populations will decrease in the same direction. If on the other hand, different eigenvalues become more “stable” for different parts of the phase space, we expect that variances of different populations might be minimized in different regions of the parameter space.

The variance analysis however provides more information. These additional insights are as follows:

- Because the size of the variance is calculated explicitly in this analysis, one can derive biologically meaningful constraints on the parameters based on the tolerable % change of a population size in a given tissue. This comes naturally from the expressions for the variance.
- From the optimization point of view, it is not clear how to weigh different eigenvalues in the linear analysis, if one were to deduce the “best” architecture. A problem arises if different eigenvalues experience minima for different parameter combinations. If explicit expressions for variances are available, then one can derive an optimization problem where the weights of different variances are controlled (see Section 3 of S1 Text for an example that is worked out in detail).
- Eigenvalues only depend on derivatives of the rates, but the variances also depend on the equilibrium values of the rates. The analysis of variance can inform us, for example, whether and by how much the magnitude of different processes affects fluctuations in each compartment.

Therefore we conclude that the study of the variances, while sharing some important features with the usual linear analysis, contributes additional information that can allow us to argue about aspects of tissue design and the functioning of stem cell lineages. In the next section we demonstrate the power of this methodology by using the example of the airway epithelium.

## Results

### The airway epithelium: Biological information

Airway epithelium lines the inner surface of the trachea and bronchi in the lung. It is organized as a two-dimensional sheet of cells sitting on top of the basement membrane. Because all cells are attached to the basal membrane, it is technically a single-layered epithelium. Its lineage consists of three principle cell types: (1) stem cells (SCs), and two distinct differentiated cell types: (2) secretory (SecrCs) and (3) ciliated cells (CilCs) [65–67]. At homeostasis all cells are distributed at the following approximate stem-to-secretory-to-ciliated ratio: 30%-15%-55%.

In terms of lineage control, the available experimental data suggests that most of the control mechanisms are autonomous, i.e. to a significant extent SCs, SecrCs and CilCs regulate each other’s dynamics. Importantly, additional regulatory signals can also come from the fibroblasts and immune cell types located beneath the basal membrane [65]. In this work, however, we will focus on the autonomous lineage controls. Airway epithelium demonstrates the following types of lineage behavior: (i) SC quiescence vs. activation, (ii) SecrCs differentiation into SecrCs or CilCs, (iii) SecrCs de-differentiation into SCs, and (iv) trans-differentiation of SecrCs into CilCs. Moreover, it is established that SecrCs can undergo proliferation, while CilCs are considered post-mitotic and their half-life is around 150 days. Most of the above behaviors can be activated upon lineage injury, when one or several cell types are depleted and the lineage repairs toward restoring homeostasis. Below we will outline three distinct previously reported airway epithelium injury experiments, the types of lineage responses that they invoke, as well as the types of regulatory mechanisms that they reveal.

#### Scenario I: Depletion of ciliated cells [27]

Surprisingly, when CilCs only are depleted using the Cre-lox genetic strategy, the remaining cells, SCs and SecrCs, do not undergo compensatory proliferative activation and differentiation (for SCs) or trans-differentiation (for SecrCs) into new CilCs to quickly compensate for the loss. Instead, very slow replenishment of the CilCs takes place, most likely at the rate not significantly different from the normal, homeostatic rate. This experiment suggests that:

(A) CilSc do not provide negative feedback to SCs and SecrCs, since the latter do not activate in response to CilC depletion.

#### Scenario II: Depletion of SCs [26]

When SCs are depleted from the airway epithelium, this results in the following responses:

- SecrCs exit quiescence and undergo proliferative activation and multiply.
- They also rapidly de-differentiate into new SCs, restoring them.
- Furthermore, they convert into more CilCs.
- Preexisting CilCs do not appear to activate, consistent with the notion that they are terminally differentiated and post-mitotic.

These observations show that, after SC depletion, 8% of SecrC progeny convert to new SCs and 34% into new CilCs, while the remainder stays as SecrCs. At that point, repaired lineage appears to equilibrate and return back to homeostasis. This experiment reveals the following additional information about the airway epithelium lineage controls:

(B) SCs provide forward control to SecrCs, maintaining them in the quiescent state (i.e. prevent proliferation);

(C) Upon homeostasis, SCs also prevent SecrCs from converting into SCs and CilCs via de-differentiation and trans-differentiation routes respectively.

In other words, SCs signal to maintain SecrC quiescence and identity. Additional experiments showed that the molecular identity of this forward SC-to-SecrC control is a short-range Notch signaling. It operates via cell-to-cell contact between neighboring cells.

#### Scenario III: Simultaneous depletion of SecrCs and CilCs [28]

When both differentiated cell types are depleted, the remaining SCs undergo rapid proliferative activation and differentiation into new CilCs and SecrCs, quickly restoring the lineage. This experiment reveals the following additional information about the airway epithelium lineage controls:

(D) Upon homeostasis, differentiated cells provide negative feedback to SCs, preventing their activation.

Considering that SCs do not become activated upon depletion of CilCs only (see scenario I), it can be assumed that most of the negative feedback is exerted by SecrCs.

### Modeling and stochastic analysis

**Fig 1:**
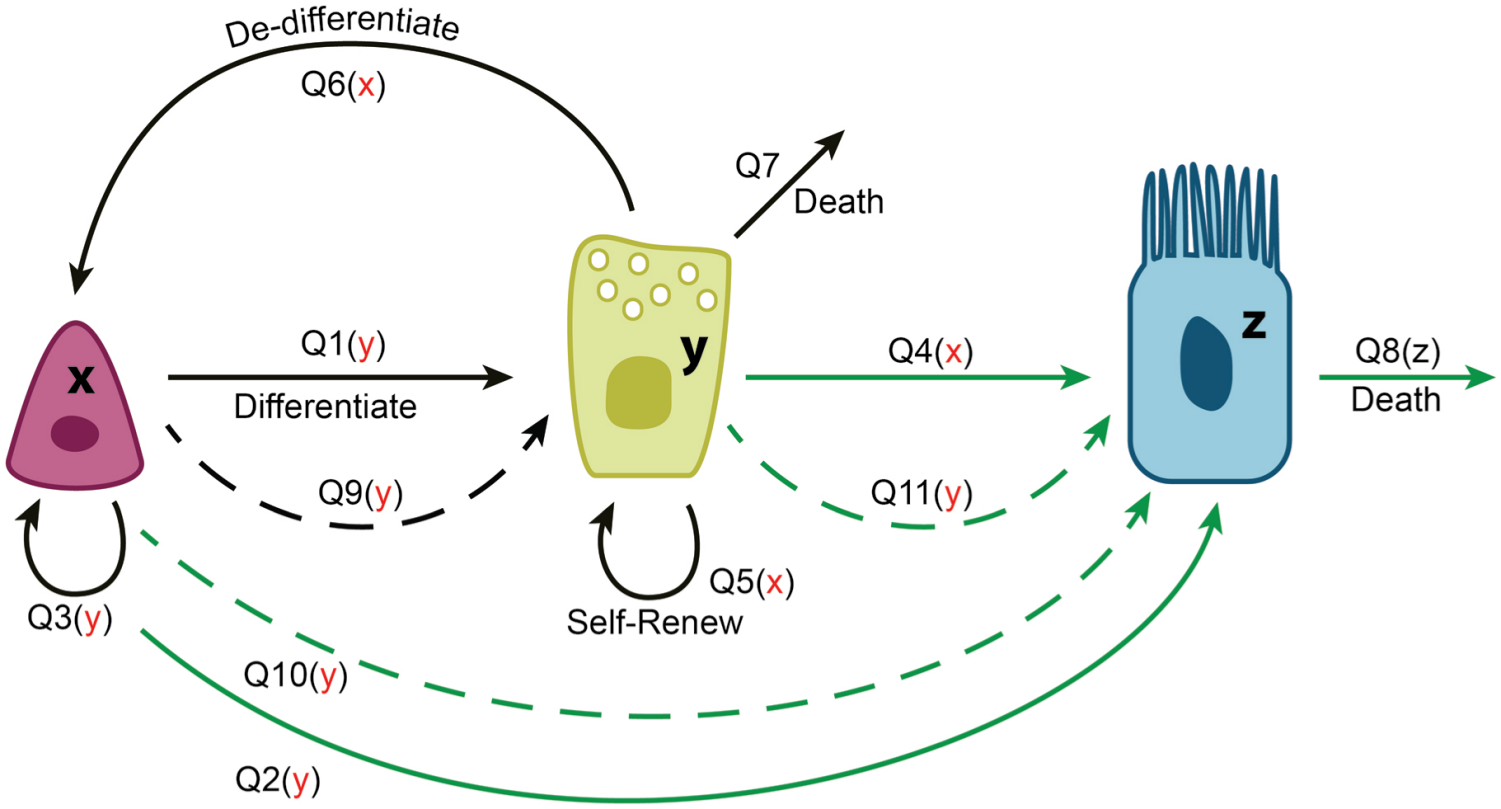
A schematic showing the cellular processes in the model of airway epithelium. The three types of cells are depicted (from left to right): SCs, SecrCs, and CilCs. They are denoted by the variables *x*, *y*, and *z* respectively. The processes are shown by arrows, where dashed lines denote asymmetric divisions, see also Table 1. For each process, its regulation, if any, is shown in the brackets, with red symbols denoting negative regulation. Processes that contribute to the slow dynamics of CilCs are denoted by greed arrows.

Below we will demonstrate the application of the modeling methodology developed here in the context of the airway epithelium regulation. In particular, we will show how the equilibrium rates are constrained, perform the stability analysis, and calculate variances. Analysis of stability and fluctuation magnitudes will allow us to argue about possible control network architectures compatible with the biological observations, and to explain the observed preferences for division symmetries and de-differentiation strategies. In Fig 1 and Table 1 we show eleven cellular processes that can happen in the airway epithelium. Each of these processes results in a change in the abundance of at least one of the three cell types, SCs, SecrCs, and CilCs. Controls are incorporated by assuming that the rate of each of the processes can be influenced by any of the existing population, such that near the equilibrium,

$$Q_{k}\left(x,y,z\right)\approx Q_{k,0}+Q_{kx}\left(x-x_{0}\right)+Q_{ky}\left(y-y_{0}\right)+Q_{kz}\left(z-z_{0}\right),$$ (8)

where (*x*_0_, *y*_0_, *z*_0_) are the equilibrium numbers of SCs, SecrCs, and CilCs respectively, *Q*_*k*0_ is the rate of process *Q*_*k*_ at equilibrium, and quantities *Q*_*kx*_, *Q*_*ky*_, *Q*_*kz*_ (which we call “controls”) are derivatives of this rate with respect to the three population sizes. These three quantities describe how strongly, and in which direction, the intensity of a process changes if each of the populations experiences a fluctuation. A negative value of such a derivative corresponds to a negative control loop.

**Table 1:** Cellular processes in the airway epithelium model.

*Q*_*k*_	Process	Δ_*k*_ *i*^1^	Δ_*k*_ *i*^2^	Δ_*k*_ *i*^3^
*Q*_1_	Differentiation of SCs into SecrCs by symmetric divisions	-1	2	0
*Q*_2_	Differentiation of SCs into CilCs by symmetric divisions	-1	0	2
*Q*_3_	Symmetric self-renewal of SCs	1	0	0
*Q*_4_	Differentiation of SecrCs into CilCs by symmetric divisions	0	-1	2
*Q*_5_	Symmetric self-renewal of SecrCs	0	1	0
*Q*_6_	De-differentiation of SecrCs	1	-1	0
*Q*_7_	Death of SecrCs	0	-1	0
*Q*_8_	Death of CilCs	0	0	-1
*Q*_9_	Asymmetric divisions of SCs producing one SecrC offspring	0	1	0
*Q*_10_	Asymmetric divisions of SCs producing one CilC offspring	0	0	1
*Q*_11_	Asymmetric divisions of SecrCs producing one CilC offspring	0	0	1

A description of the processes depicted in Fig 1. Although processes *Q*_5_ and *Q*_9_, *Q*_10_ and *Q*_11_ are equivalent from the viewpoint of cellular population change, we count them as different processes, because they can be regulated differently.

To be precise, paper [29] identified more complexity in the dynamics of SCs in the airway epithelium. It was found that SCs do not divide directly into CilCs or SecrCs. Instead, they create (by predominantly asymmetric divisions) a different type of progenitor cell (called luminal progenitors) which later mature into SecrCs. Our model combines this into just one step, an asymmetric division into SecrCs. Adding this intermediate step effectively changes the rate of process *Q*_9*y*_ and is therefore not implemented.

Although each of the controls may be a nontrivial number, we strive to create the simplest model that is compatible with the existing observations. Such a model must include a negative regulation of SC divisions by SecrCs (fact **(D)** above). Further, divisions and de-differentiation of SecrCs is negatively regulated by SCs (facts **(B), (C)** above). Finally, CilCs do not exert any known control over the processes happening in the SC and SecrC compartments (fact **(A)**). We further assumed that the overall rate of CilC death increases with their abundance (note that this is not a per-cell rate, but the overall intensity of apoptosis). Therefore, only some of the derivatives in eq (8) will be nonzero. We list these possible controls here:

$$Q_{1y},Q_{2y},Q_{3y},Q_{4x},Q_{5x},Q_{6x},Q_{8z},Q_{9y},Q_{10,y},Q_{11,y}.$$ (9)

All of these are nonpositive except *Q*_8*z*_, which is nonnegative.

Interestingly, not all of the eleven processes make equal contribution to the maintenance of stable homeostasis. In [29], it was shown that an overwhelming majority of SC divisions are asymmetric, and an overwhelming majority of the SecrC divisions are symmetric. Our first goal is to explain this type of design.

#### Why do airway epithelium SecrCs divide mostly symmetrically?

Let us employ formulation (8) (where the equilibrium cell numbers is consistent with experimentally measured values [67–69], (250, 200, 550)), and then use system (2) to define three constraints on the unknown equilibrium process rates:

$$Q_{8,0}=2Q_{2,0}+2Q_{4,0}+Q_{10,0}+Q_{11,0},$$ (10)

$$2Q_{5,0}=-2Q_{1,0}-2Q_{3,0}+2Q_{7,0}+Q_{8,0}-2Q_{9,0}-Q_{10,0}-Q_{11,0},$$ (11)

$$Q_{6,0}=Q_{1,0}+Q_{2,0}-Q_{3,0}.$$ (12)

We will use the experimental fact on the *rates* of divisions to argue about the *symmetries* of divisions. It has been reported in [27] that the production/death dynamics of the airway epithelium CilCs are significantly slower than that of SCs and SecrCs. From Fig 1 it is apparent that the processes that change the number of CilCs are *Q*_2_, *Q*_4_, *Q*_8_, *Q*_10_, *Q*_11_ (they are marked by green arrows on the figure). To maintain a constant level of CilCs, they have to balance out in the way described by eq (10). In particular, a known low equilibrium death rate of CilCs, *Q*_8,0_ means that all the division rates for processes *Q*_2_, *Q*_4_, *Q*_10_, *Q*_11_ will be similarly low. Assuming that the turnover rates of SCs and SecrCs are higher than those for CilCs, we immediately obtain that of the three division processes for SecrCs (*Q*_5_, *Q*_4_, *Q*_11_), the symmetric proliferation must be the highest. In other words, SecrCs are predicted to divide predominantly in a symmetric way by an argument simply based on cell balance at the equilibrium.

#### Why do airway epithelium SCs divide mostly asymmetrically?

Based on the above argument, by which *Q*_2_, *Q*_4_, *Q*_8_, *Q*_10_, *Q*_11_ are all slow processes, we can simplify the description of the system’s dynamics. Namely, we separate the time-scales and consider the relatively fast dynamics of SCs and SecrCs separately, as a two-compartment system. This is achieved by assuming that the population of CilCs changes slowly (and is a constant on the time-scale of the change in SCs and SecrSc). Because no processes involving SCs or SecrCs are regulated by CilCs, the equations for SCs and SecrCs separate. In this system, the following processes take place:

$$Q_{1},Q_{3},Q_{5},Q_{6},Q_{9}.$$

We would like to understand why SCs tend to divide predominantly in an asymmetric way, by looking at the variances of the cell populations. Which relative values of *Q*_1_ and *Q*_3_ can minimize the variance?

We begin by rewriting eqs (11) and (12) to include only nonzero equilibrium values:

$$Q_{5,0}=-Q_{1,0}-Q_{3,0}+Q_{7,0}-Q_{9,0},$$ (13)

$$Q_{6,0}=Q_{1,0}-Q_{3,0}$$ (14)

(the first equation, Eq (10), is now an identity 0 = 0). Further, we examine the expressions for the variance, by solving eq (6), see S1 Text, Section 5 for the relevant expressions. It turns out that the variances satisfy the following properties:

- The variance of CilCs is zero, because in the current approximation CilC dynamics is not included: *Var*[*z*] = 0.
- The variances of SCs and SecrCs, *Var*[*x*] and *Var*[*y*], do not depend on the amplitude *Q*_9,0_ of asymmetric SC divisions.
- They are linear functions of the amplitudes *Q*_1,0_ and *Q*_3,0_ of symmetric SC divisions, with coefficients that depend of the controls.

Therefore, we can reduce our optimization problem to that of linear minimization, see S1 Text, Section 5 (simple linear programming techniques work for a subclass of systems, and in the most general case a proof is provided based on the matrix properties of solution Eq (7)). It turns out that both SC and SecrC variances are minimized for zero values of *Q*_1,0_ and *Q*_3,0_, which in turn suggests that the optimal SC division pattern from the point of view of homeostasis maintenance is asymmetric divisions. Incidentally, asymmetric SC divisions are also associated with a higher degree of robustness of the system: the stability condition in the absence of SC symmetric divisions becomes *Q*_6*x*_ *Q*_9*y*_ > 0, and is always satisfied under the correct sign assignments for the controls.

#### Stability and recovery dynamics

**Fig 2:**
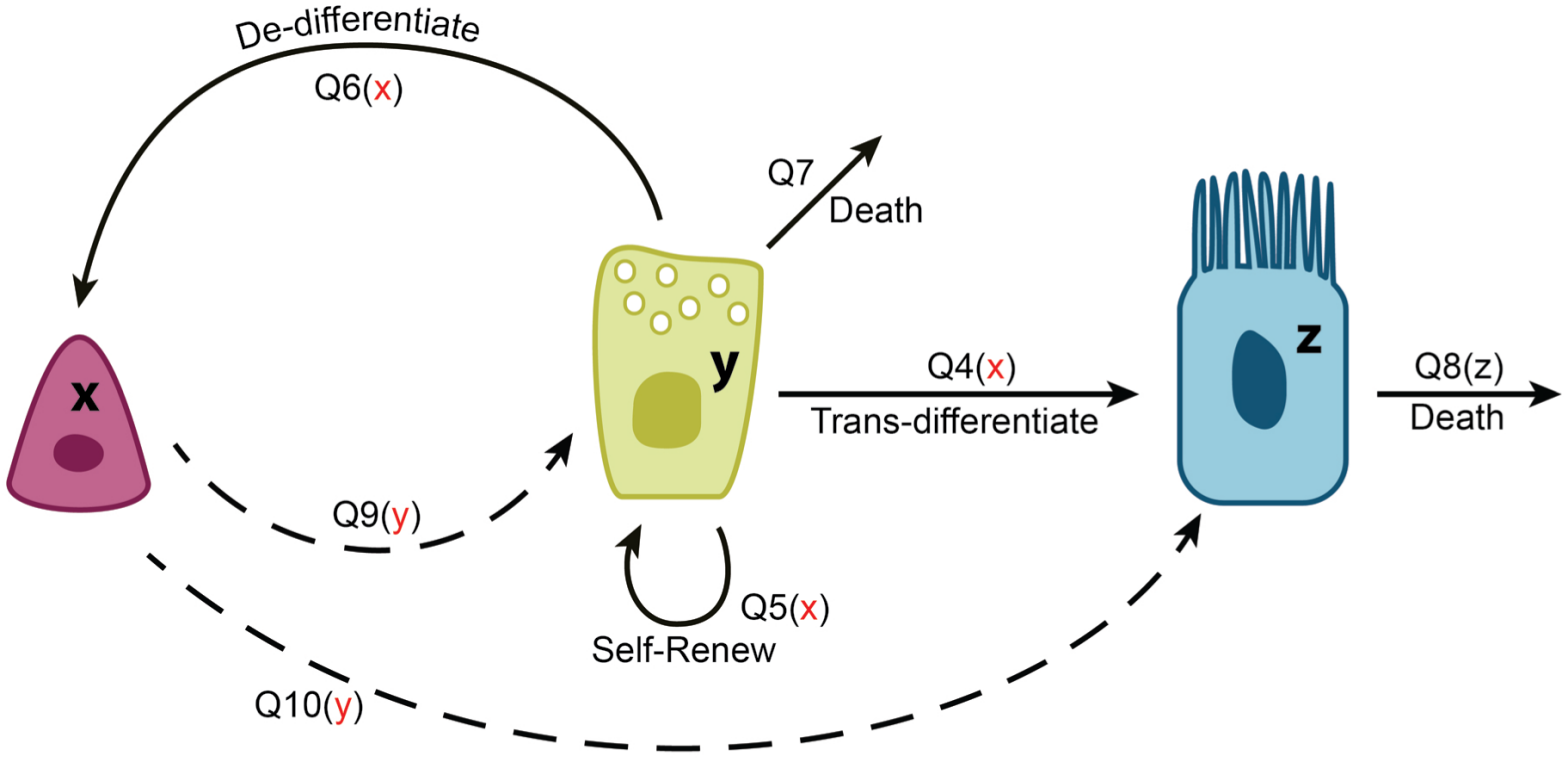
A schematic showing the cellular processes in the reduced model of airway epithelium. *x* denotes SCs, *y* denotes SecrCs, and *z* denotes CilCs. SCs only divide asymmetrically, and SecrCs only divide symmetrically.

Given the above considerations and observations of [29], we will assume that SCs divide purely asymmetrically and SecrCs divide purely symmetrically, by setting *Q*_1_ = *Q*_2_ = *Q*_3_ = *Q*_9_ = *Q*_11_ = 0, see Fig 2. Other possibilities are explored in S1 Text, Section 4, where it is demonstrated that under assumptions **(A)-(D)**, the system is fairly robust, and a number of other combinations of processes, such as all symmetric divisions, all asymmetric divisions, or mixed divisions, can lead to stable homeostasis.

Systems (10)–(12) can be rewritten as

$$Q_{6,0}=0,$$ (15)

$$Q_{9,0}=Q_{4,0}-Q_{5,0}+Q_{7,0},$$ (16)

$$Q_{10,0}=-2Q_{4,0}+Q_{8,0}.$$ (17)

Note that the first of these equations suggests that under homeostatic conditions, de-differentiation of SecrCs is suppressed, which coincides with the results of [29]. To analyze stability, we can find the eigenvalues of the Jacobian Eq (3), which in this case are simply

$$Q_{6x},Q_{5y}+Q_{9y},-Q_{8z},$$ (18)

all negative numbers under our assumptions. This demonstrates that the model is extremely robust, in the sense that any numerical values of the controls with the correct sign will guarantee stability. Here we used the term “robust” in the same was as we did in [5], that is, the system is stable under a large parameter set.

To find the variance, we solve eq (6). In the case of the control system depicted in Fig 2, we find

$$\begin{array}{lll} y_{11} & = & 0,\\ y_{22} & = & \frac{Q_{4,0}+Q_{5,0}+Q_{7,0}}{2|Q_{9y}|},\\ y_{33} & = & \frac{Q_{8,0}Q_{9y}(Q_{9y}-Q_{8z})+Q_{4,0}\left[{\left(2Q_{9y}+Q_{10y}\right)}^{2}-4Q_{9y}Q_{8z}\right]+(Q_{5,0}+Q_{7,0})Q_{10y}^{2}}{2Q_{9y}Q_{8z}(Q_{9y}-Q_{8z})}. \end{array}$$ (19)

**Fig 3:**
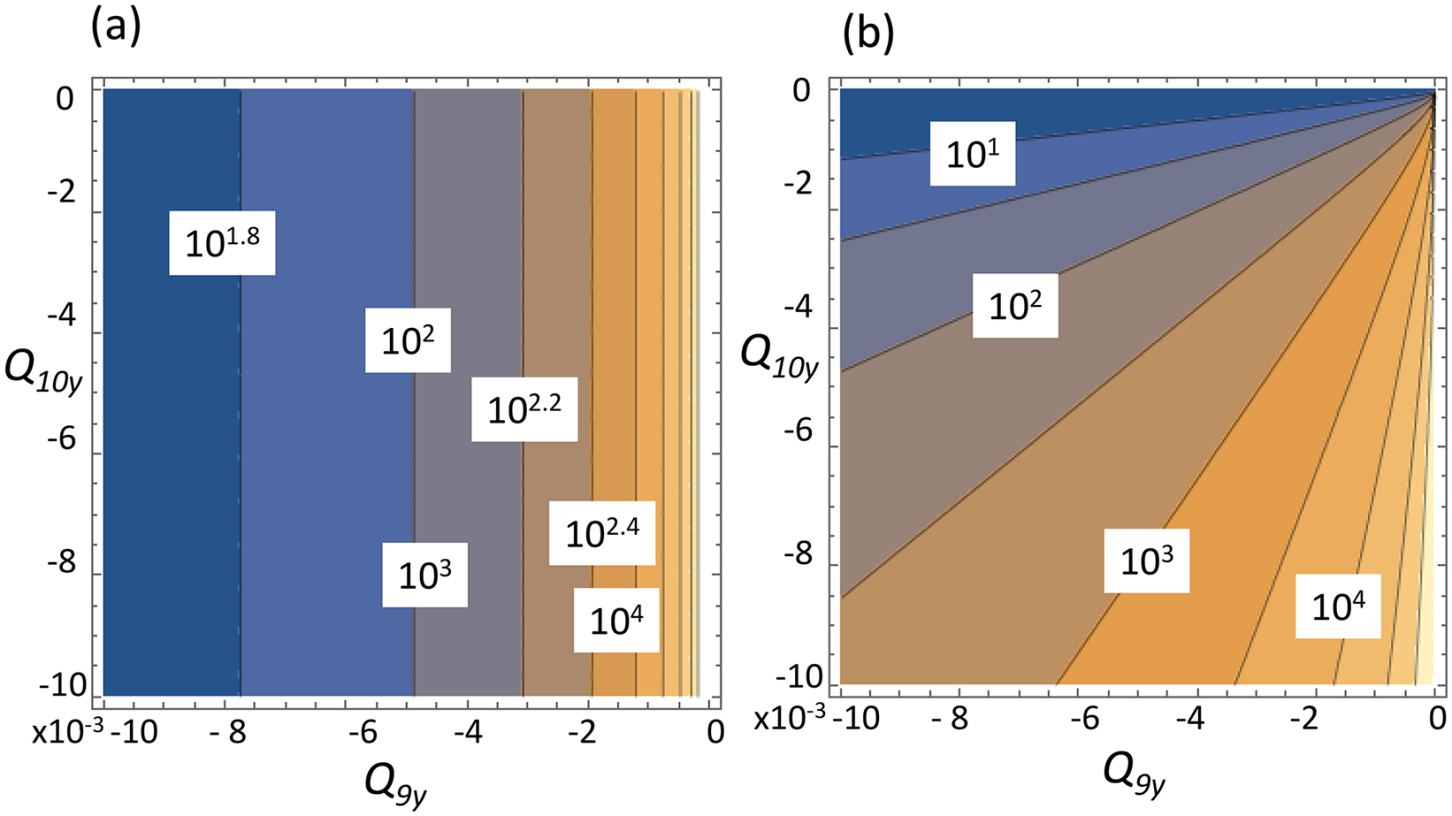
The variance of (a) SecrCs and (b) CilCs as a function of system’s parameters. Two controls are varied: *Q*_9*y*_ measures the strength of inhibition of SC differentiation into SecrCs, and *Q*_10*y*_ measures inhibition of differentiation into CilCs. The contour plots have the levels marked. The rest of the parameters are *Q*_4_ = 0.00078 − 0.0074(*x* − *x*_0_),*Q*_5_ = 0.041 − 0.0058(*x* − *x*_0_),*Q*_6_ = 0.32 − 0.0043(*x* − *x*_0_),*Q*_7_ = 0.937,*Q*_8_ = 0.0056 + 0.001(*z* − *z*_0_),*Q*_9_ = 0.0057 + *Q*_9*y*_(*y* − *y*_0_),*Q*_10_ = 0.467 + *Q*_10*y*_(*y* − *y*_0_); *x*_0_ = 250, *y*_0_ = 200, *z*_0_ = 550.

The first equation suggests that the number of SCs in this system does not vary; this is because SCs only divide asymmetrically and their numbers do not change. The second and third equations present the variance of SecrCs and CilCs in terms of system parameters. Fig 3 shows how these two quantities depend on the amount of inhibition of SC divisions by the SecrCs. We can see that a strong inhibition of differentiation into SecrCs, and a weak inhibition of differentiation into CilCs corresponds to the smallest variance.

The next observation concerns the concept of “minimal control” [5] (a minimal control is a control network with the smallest possible number of loops). What control loops can be eliminated from the system without compromising its stability? Further, elimination of which controls does not increase the system’s variance? Quantities *Q*_4*x*_ and *Q*_10*y*_ do not enter the stability condition; *Q*_4*x*_ does not change the variances, and decreasing |*Q*_10*y*_| helps to minimize the variance of CilCs. These observations lead us to conclude that the negative control of SC asymmetric divisions into CilCs and differentiation of SecrCs into CilCs do not have to be under control from any of the cell populations. Eliminating those control loops (that is, setting *Q*_10*y*_ = *Q*_4*x*_ = 0), does not make the system unstable. In fact, it helps to decrease the variance of CilCs without affecting the variance of other cell types.

Finally, we examine the dynamics of the cell populations under control system in Fig 2. Because we do not have numerical values for the coefficients, we have explored a large number of systems where coefficients were generated randomly. First, we generated coefficients *Q*_5,0_ and *Q*_7,0_ as uniformly distributed random numbers between 0 and 1. Then, we made sure that the steady-state death rate of the CilCs is small (as suggested by measurements), and generated *Q*_4,0_ and *Q*_8,0_ as random numbers in [0, *ϵ*] (with *ϵ* = 0.01). The rest of the steady state values are given by eqs (15)–(17); we only selected combinations that resulted in positive values for these coefficients. Next, we generated the negative controls *Q*_4*x*_,*Q*_5*x*_,*Q*_6*x*_,*Q*_9*y*_,*Q*_10*y*_ in the range [−*ϵ*, 0], and the positive control *Q*_8*z*_ in the range [0, *ϵ*]. Any system created in this way will be stable because of the form of the eigenvalues Eq (18).

**Fig 4:**
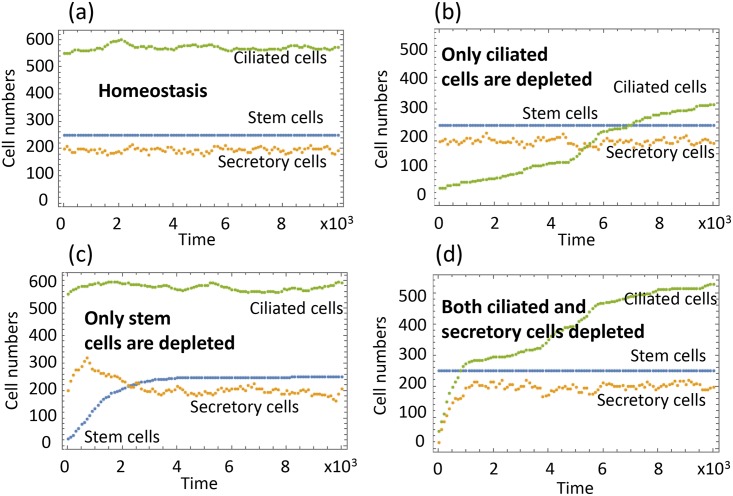
Homeostasis and recovery dynamics in the airway epithelium. Typical runs with different initial conditions are presented. (a) Homeostasis: the initial condition is given by *x* = *x*_0_, *y* = *y*_0_, *z* = *z*_0_. (b) Experimental scenario I: the initial condition is given by *x* = *x*_0_, *y* = *y*_0_, *z* = 0.1 *z*_0_. (c) Scenario II: *x* = 0.1 *x*_0_, *y* = *y*_0_, *z* = *z*_0_. (d) Scenario III: *x* = *x*_0_, *y* = 0.1 *y*_0_, *z* = 0.1 *z*_0_. The equilibrium values are as in Fig 3, and the rest of parameters are: *Q*_4_ = 0.0089 − 0.0008(*x* − *x*_0_),*Q*_5_ = 0.083 − 0.0092(*x* − *x*_0_) − 0.006(*y* − *y*_0_),*Q*_6_ = 0.85 − 0.0022(*x* − *x*_0_),*Q*_7_ = 0.911,*Q*_8_ = 0.0019 + 0.0029(*z* − *z*_0_),*Q*_9_ = 0.0075 − 0.0027(*y* − *y*_0_),*Q*_10_ = 0.66 − 0.0094(*y* − *y*_0_);

In Fig 4(a) one can see a typical run of the system under homeostasis. In stochastic simulations, at each update, exactly one cellular process happens out of the possibilities in Fig 2. The probability of process *k* is given by *Q*_*k*_/∑_*m*_ *Q*_*m*_. The functions *Q*_*k*_(*x*, *y*, *z*) are assumed piecewise linear. They are given by expression (8) if it is positive, and they are equal to zero otherwise.

Fig 4(b)–4(d) show recovery dynamics, reproducing experimentally tested scenarios (I-III) described above. In (a), we start with a 90% reduced population of CilCs, and observe a very slow recovery, as demonstrated in scenario (I). This is a slow process because the processes maintaining CilCs are slow (due to the smallness of the coefficients in the functions *Q*_8_ (death) and *Q*_10_, *Q*_4_ (replenishment by SCs and SecrCs respectively). The intensity of these processes does not change as CilCs are depleted. The recovery happens purely by breaking the balance between deaths (which are less frequent if CilCs are depleted) and production (which does not change as CilCs are removed).

Fig 4(d) demonstrates scenario (III), where both CilCs and SecrCs are depleted. We can see a relatively fast recovery of both cell types. In particular, when SecrCs are depleted, the decrease in *y* upregulates the activity of SCs, which differentiate into SecrCs thus replenishing both SecrCs and CilCs, consistent with the reported biological observations.

Fig 4(d) demonstrates scenario (II), where SCs are depleted, and then successfully recover. This is an interesting case given that under homeostatic conditions the population of SCs remains constant. If it is depleted, the SecrCs are triggered into de-differentiation, providing a mechanism of recovery for SCs.

## Discussion

In this work we studied stochastic multi-compartment dynamics of SCs and their lineages. We developed a simple and effective method to mathematically describe any type of multi-compartment lineage system. We could find the analytical results for the expectation and variance of the population of any type of lineage-connected cells, assuming that we know the inverse of a simple deterministic matrix. Furthermore, the stability conditions for the multi-compartment SC dynamics were identified.

The general method developed in this paper is applicable for studying a very large class of cellular lineages, and not just simple linear *n*-compartment models. The technique can naturally include any type of hierarchical or two-way relationships among cells. As described in the Introduction, in most tissues and organs, there are more than just two types of cells, and hierarchical cellular networks are sometimes arranged in a complex nonlinear fashion. Our technique (and the symbolic algorithm developed) are capable of handling such systems. The goal is to study the stability and robustness of control networks that maintain homeostasis in such complex systems.

We also applied these techniques to interrogate the lineage dynamics in a particular biological system, the mouse airway epithelium. In this system, there are three principal types of cells (SCs, SecrCs, and CilCs) that are lineage-connected and can influence each other’s fate decisions. Symmetric and asymmetric divisions, deaths, differentiation and de-differentiation can all take place. There are significant available biological data with regards to the division types and the recovery dynamics in response to injury that take place in the airway epithelium, thus enabling us to compare mathematically derived behaviors with the actual cellular actions. Interestingly, we found that there are multiple ways in which all of the above cellular processes can be mathematically arranged and regulated so that they are stable and compatible with the existing experimental evidence on the lineage recovery dynamics. For example, mathematically, all divisions can be symmetric, or asymmetric, or mixed, and either one of these division types is compatible with the biologically observable lineage dynamics. Yet, recent biological data shows that airway epithelium SCs divide almost exclusively asymmetrically, while SecrCs divide almost exclusively symmetrically.

By using the framework developed here, we offer an explanation of these symmetry patterns. It turns out that the predominantly symmetric divisions of SecrCs can be ascribed to the requirement of balance of various cellular processes at equilibrium. At equilibrium, one must expect to have cellular loss (from say differentiation, de-differentiaion, or death) to be balanced by cellular gain. In the case of the airway epithelium, we considered the peculiarly slow turnover dynamics of CilCs and derived a balance equation for CilC change. Then, from the requirement that the death rate of CilCs is slow [70], we deduced that by necessity, SecrCs must divide predominantly symmetrically, to avoid unbalanced accumulation of CilCs.

Further we provided an explanation of the predominantly asymmetric divisions of SCs. We considered a system where both symmetric and asymmetric division types for SCs were included, and asked what arrangement of equilibrium division rates will minimize the magnitude of cell number fluctuations. It turned out that strictly asymmetric divisions of SCs comprise the optimal solution for this linear minimization problem under the given biological constraints (such as positivity of cell numbers and rates). Therefore, by using our methodology, we showed that the observed division pattern in the airway epithelium is the only one that is consistent with the steady cell numbers, slow turnover dynamics of the CilCs, and minimal variance of the cell populations at homeostasis.

We have also focused on a particular lineage behavior revealed in the recent work suggesting the lack of negative feedback from the differentiated CilCs to SCs or SecrCs following genetic depletion of CilCs [29]. We used the smallest possible number of control loops to study this phenomenon mathematically. We show that minimally parameterized model can robustly mimic the biologically observable slow CilCs recovery dynamics. Furthermore, the same model can robustly mimic quick lineage recovery dynamics when both CilCs and SecrCs are depleted. Consistent with the speculated mechanism, we now show quantitatively that robust, biologically compatible airway epithelium lineage behaviors are possible when only one out of two differentiated cell types (SecrCs) provide negative feedback to SCs. This control arrangement explains why no lineage recovery mechanism gets triggered when only CilCs are injured.

On its surface, it would appear that such lineage “blindness” to CilC depletion represents a major vulnerability of the airway epithelium. Clearly, biologically speaking, lack of quick epithelium repair would compromise its anatomical integrity and function. What can then explain this seemingly irrational control design? We hypothesize that this way of lineage control represents an example of an evolutionary “economy”. Clearly, having two negative control loops to SCs (both from the CilCs and SecrCs) would lead to a robust and quick recovery following all types of differentiated cell loss. However, in a real-life situation it is likely unnecessary for the epithelium to be able to quickly recover from the loss of only one type of differentiated cells. To-date, there are no natural events that would deplete one but not the other type of differentiated cells; this can be only induced experimentally using an artificial genetic system. On the other hand, depletion of both CilCs and SecrCs happens, commonly following inhalation of the poisonous naturally occurring sulfur dioxide (SO2) [71, 72], or as the result of acute viral infection, such as with the influenza virus [26, 73]. Therefore, such naturally occurring injuries are enough to be able to trigger repair mechanisms by removing an inhibitory signal emanated by just one cell type. Conditions of scenario III (specific CilC loss) do not represent a situation for which an organism should be prepared. This interesting experiment reveals the absence of a signaling loop from CilCs back to SCs. We can think of this arrangement of control loops as an example of cooperation among different cell types. SecrCs signal back to SCs to help recover their own loss and the loss of CilCs.

Another type of question that can be addressed with our framework is the necessity for various processes in control networks. For example, stability analysis shows that SecrCs de-differentiation to SCs in the airway epithelium is not observed under the equilibrium conditions (to keep the balance of cell numbers, eq (15), which coincides with earlier reports [29]). At the same time, de-differentiation is the process that has been experimentally shown to allow for the quick recovery of the SC numbers after their removal [26], see Fig 4(c). The question arises whether de-differentiation may have another role in the system, because catastrophic SC depletion (of the type created in the experimental setup of [26]) is probably unlikely under natural conditions. Why did the mechanism of de-differentiation evolve in the first place? The answer to this question comes directly from our theory. The presence of de-differentiation, and more specifically, de-differentiation controlled negatively by the SC population, is a necessary condition for the system’s stability, as follows from the expression for the first eigenvalue in Eq (18). The biological explanation of this condition is that SC death occurs at low rates (e.g. due to mutations). Since SCs divide strictly asymmetrically, they are not able to compensate for low rate of SC death over time. We propose that this can be compensated by SecrC de-differentiation, as follows from out analysis.

Finally we need to mention the numerous limitations of this study in particular and the methodology developed in general. The biggest drawback is the absence of spatial considerations. In the literature, spatial models of SC dynamics have been studied by several authors [41, 55, 60, 74–77], see also the reviews [78–80]. Analytical results have only been obtained in the simplest systems, and did not include any considerations of regulatory networks. Our first attempts of the analytical treatment of spatially distributed SC systems are concerned with cell mutagenesis and cancer generation [81]. In [82] we provide analytical solutions of a very simple, spatially regulated SC lineage again in the context of carcinogenesis and tumor suppressor gene inactivation. The present framework can only mimic spatial tissue organization by weighing “local” and “global” control loops differently. An explicit treatment of spatial structures is subject of future work. Another limitation of this theory is the requirement of relatively small deviations from the equilibrium. Theoretical basis for this approach (which stems from the linear noise approximation [61]) requires a weak dependence of the control functions on cell population numbers. While injury recovery dynamics certainly can be modeled by means of stochastic simulations, as we did in the current paper, the theory is inherently “local”. More analysis is required to study the global stability and global dynamics of SC systems, see [50–52].

As the final message, we would like to propose that the current framework can be used to study the general principles that govern SC lineage dynamics, across tissues. Several such candidate principles come to mind, including (1) “economy” (the non-existence of overlapping controls not needed for stability or robustness), (2) “cooperation” (such as in the example given by SecrCs signaling back to SCs to help compensate for the CilC loss as well as their own), and (3) “robustness” in the sense that certain loop arrangements allow stability for very large parameter regions, compared to others. In the airway epithelium example, the observed network is stable for any parameter values as long as they have the correct sign, in contrast to some other network configurations considered in S1 Text, Section 4. By using our methodology, one can study such patterns of cell regulation and ask how they trade off in the context of stability and variance minimization. This is one of future directions of research and immediate applications of the near equilibrium calculus of stem cells developed here, albeit in some tissue types application of this technique can be hindered by the time scale and spatial scale separation of cells within the lineages.

## Supporting Information

S1 Text An in-depth mathematical analysis, further examples, and exploration of the parameter space. Additional figures are presented.
(PDF)

S1 File A *Mathematica* file that performs the symbolic analysis of the lineage dynamics for a given model. Equilibrium conditions, stability analysis and the variance calculations are presented.
(NB) Click here for additional data file.
