# Supplementary material for: Near Equilibrium Calculus of Stem Cells in Application to the Airway Epithelium Lineage
Source: PLoS Comput Biol. 2016 Jul 18;12(7):e1004990. doi: 10.1371/journal.pcbi.1004990 (PMC4948767; doi:10.1371/journal.pcbi.1004990)
Supplement: S1 Text — Additional figures are presented. (PDF) [file pcbi.1004990.s001.pdf]

Stochastic Calculus of Stem Cells in Application To The Airway Epithelium Lineage

Zheng Sun, Maksim V. Plikus, and Natalia L. Komarova

Text S1

Contents

1 Methodology **2**
- 1.1 A general two-compartment model 2
  - 1.1.1 The equilibrium state and the deterministic description for the two-compartment model 2
  - 1.1.2 Stochastic analysis of the two-compartment model . . . 4
- 1.2 A general multi-compartment model 6
  - 1.2.1 Stochastic analysis of the multi-compartment model . . 6

2 Examples **9**
- 2.1 A two-compartment model 9
- 2.2 A 3-compartment system with negative control of divisions and differentiation 10
- 2.3 A 3-compartment system with positive control of differentiation and negative control of divisions 14

3 Stability and variance calculations **16**

4 Mouse airway epithelium: examples of networks, their stability, and recovery dynamics **19**
- 4.1 Only symmetric divisions 21
- 4.2 Only asymmetric divisions 24

5 Minimization of variance as a function of the equilibrium rates **25**
- 5.1 Assumption of the smallness of derivatives 26
- 5.2 General proof 28

1 Methodology

1.1 A general two-compartment model

We consider a two-compartment model consisting of SCs and differentiated cells. We will refer to the number of stem cells as i and to the number of differentiated cells as j. Suppose further the existence of several (K) processes that may change the numbers of cells. Each process is characterized by the associated change in cell numbers, $(\Delta_k i, \Delta_k j)$, $1 \leq k \leq K$. For example, the process of proliferation of a SC increases the number of SCs by one and does not change the number of differentiated cells. The process of differentiation decreases the number of SCs by one and increases the number of differentiated cells by two. The process of differentiated cell death decreases j by 1. These are the three processes that we considered in [1]. In the present formalism, this amounts to $K = 3$ nontrivial transition probabilities summarized below:

$$\begin{aligned} Q_1: &\quad \text{Proliferation of SCs}, \quad \Delta_1 i = 1, \quad \Delta_1 j = 0, \\ Q_2: &\quad \text{Differentiation of SCs}, \quad \Delta_2 i = -1, \quad \Delta_2 j = 2, \\ Q_3: &\quad \text{Death of differentiated cells}, \quad \Delta_3 i = 0, \quad \Delta_3 j = -1. \end{aligned} \tag{1}$$

Other possible processes include a SC death ($\Delta i = -1$, $\Delta j = 0$); an asymmetric SC division ($\Delta i = 0$, $\Delta j = 1$), a de-differentiation event ($\Delta i = -1$, $\Delta j = 1$) etc.

We assume that the dynamics of cells is governed by a continuous-time Markov process characterized by transition probabilities $Q_k(i,j)$ associated with the K processes. In an infinitely small time interval, Δt, the provability that the cell numbers will change by $(\Delta_k i, \Delta_k j)$ is given by $Q_k(i,j)\Delta t$, for $1 \leq k \leq K$, and the probability of no change is given by $1 - \Delta t \sum_{k=1}^{K} Q_k(i,j)$.

1.1.1 The equilibrium state and the deterministic description for the two-compartment model

Let us assume the existence of an equilibrium state, (i_*, j_*) characterized by

$$\sum_{k=1}^{K} Q_k(i_*, j_*)\Delta_k i = \sum_{k=1}^{K} Q_k(i_*, j_*)\Delta_k j = 0.$$

In what follows, we will use the notation

$$Q_{k*} = Q_k(i_*, j_*), \quad 1 \le k \le K.$$

The phenomenological equations for the mean numbers, $x = \langle i \rangle$, and $y = \langle i \rangle$ read,

$$\dot{x} = \sum_{k=1}^{K} Q_k(x,y)\Delta_k i, \tag{2}$$

$$\dot{y} = \sum_{k=1}^{K} Q_k(x,y)\Delta_k j, \tag{3}$$

where we replaced $Q_k(i,j) \to Q_k(x,y)$. Such equations are commonly used in deterministic modeling of SCs (see e.q. [2]). They can be derived from the stochastic process by using the linear noise approximation of Van Kampen, where they appear as the zero-order expansion and comprise the "macroscopic law".

The stability analysis of the equilibrium state, $x = i_*$, $y = j_*$ results in the Jacobian,

$$J = \begin{pmatrix} a_{11} & a_{12} \\ a_{21} & a_{22} \end{pmatrix},$$

where

$$a_{11} = \sum_{k=1}^{K} \frac{\partial Q_k}{\partial x}\Delta_k i, \quad a_{12} = \sum_{k=1}^{K} \frac{\partial Q_k}{\partial y}\Delta_k i,$$
$$a_{21} = \sum_{k=1}^{K} \frac{\partial Q_k}{\partial x}\Delta_k j, \quad a_{22} = \sum_{k=1}^{K} \frac{\partial Q_k}{\partial y}\Delta_k j.$$

The stability conditions are

$$Det(J) > 0 \Leftrightarrow a_{11}a_{22} - a_{12}a_{21} > 0, \tag{4}$$

$$Tr(J) < 0 \Leftrightarrow a_{11} + a_{22} < 0. \tag{5}$$

The deterministic analysis is not capable of informing us about the variance of the cell numbers. In order to find out how big the spread of values is, we need to perform stochastic analysis detailed next.

1.1.2 Stochastic analysis of the two-compartment model

For convenience, let us use the notation i and j for the difference between the actual number of SCs and differentiated cells and their equilibrium values (such that at equilibrium, $i = j = 0$). Based on the transition probabilities, one can write down the Kolmogorov forward equations for the probability $\varphi_{i,j}(t)$ to be in the state (i, j) at time t:

$$\dot{\varphi}_{i,j} = \sum_{k=1}^{K} \varphi_{i-\Delta_k i, j-\Delta_k j}(t) Q_k(i - \Delta_k i, j - \Delta_k j) - \varphi_{i,j}(t) \sum_{k=1}^{K} Q_k(i, j). \quad (6)$$

Let us use the following notation for the moments:

$$x_{\alpha,\beta}(t) = \sum_{i,j} \varphi_{i,j}(t) i^\alpha j^\beta.$$

We can derive equations for moments by multiplying equation (6) by $i^\alpha j^\beta$ and summing up in both indices. We have at order (α, β):

$$\dot{x}_{\alpha,\beta} = \sum_{k=1}^{K} \sum_{i,j} \varphi_{i,j}(t) Q_k(i, j)(i + \Delta_k i)^\alpha (j + \Delta_k j)^\beta - \sum_{k=1}^{K} \sum_{i,j} Q_k(i, j) i^\alpha j^\beta.$$

In these equations, we will use the binomial formulas:

$$(i + \Delta_k i)^\alpha = \sum_{r=0}^{\alpha} \binom{\alpha}{r} i^r (\Delta_k i)^{\alpha-r}, \quad (7)$$

$$(j + \Delta_k j)^\beta = \sum_{s=0}^{\beta} \binom{\beta}{s} j^s (\Delta_k j)^{\beta-s}. \quad (8)$$

Near the equilibrium we can expand the probability rates $Q_k(i, j)$ in Taylor series, keeping only the terms up to the first order:

$$Q_k(i, j) = Q_{k*} + \frac{\partial Q_{k*}}{\partial x} i + \frac{\partial Q_{k*}}{\partial y} j,$$

where the two partial derivatives are taken at the equilibrium. The linear noise approximation [3] can be obtained by assuming that probability rates depend weakly on their variables, which means that the change in a given rate caused by adding or removing one cell is small relative to the rate itself.

The details are given in [1] and also [4], which suggests a simpler way of deriving the equations for the moments. We obtain the following system:

$$\dot{\mathbf{x}} = A\mathbf{x} + F = \vec{0}, \tag{9}$$

where

$$\mathbf{x} = (x_{10}, x_{01}, x_{20}, x_{02}, x_{11})^T,$$
$$F = (0, 0, s_{ii}, s_{jj}, s_{ij})^T$$

with

$$s_{ii} = \sum_{k=1}^{K} Q_{k*}(\Delta_k i)^2 \geq 0, \quad s_{jj} = \sum_{k=1}^{K} Q_{k*}(\Delta_k j)^2 \geq 0, \quad s_{ij} = \sum_{k=1}^{K} Q_{k*}\Delta_k i \Delta_k j.$$

The matrix A is given by

$$A = \begin{pmatrix} a_{11} & a_{12} & 0 & 0 & 0 \\ a_{21} & a_{22} & 0 & 0 & 0 \\ * & * & 2a_{11} & 0 & 2a_{12} \\ * & * & 0 & 2a_{22} & 2a_{21} \\ * & * & a_{21} & a_{12} & a_{11} + a_{22} \end{pmatrix}.$$

The entries marked by stars are unimportant for the stability analysis because of the block structure of the matrix A. Let us denote

$$\Delta = Det(J) = a_{11}a_{22} - a_{12}a_{21}, \quad B = -Tr(J) = -a_{11} - a_{22}.$$

We can see that at equilibrium, $x_{10} = x_{01} = 0$, and

$$Var[i] = x_{20} = \frac{1}{2B\Delta}\left(s_{ii}\Delta + s_{ii}a_{22}^2 - 2s_{ij}a_{21}a_{22} + s_{jj}a_{21}^2\right), \tag{10}$$
$$Var[j] = x_{02} = \frac{1}{2B\Delta}\left(s_{jj}\Delta + s_{jj}a_{11}^2 - 2s_{ij}a_{12}a_{11} + s_{ii}a_{12}^2\right). \tag{11}$$

The stability conditions for this equilibrium are the same as (4-5), because the eigenvalues of the matrix W are given by $-B$ and $-B \pm \sqrt{B^2 - 4\Delta}$, where the matrix W is the the submatrix $A_{\{3,4,5\},\{3,4,5\}}$.

Condition on the positivity of the variances are obtained from equations (10-11) and are given by

$$2s_{ij}a_{21}a_{22} < s_{ii}(\Delta + a_{22}^2) + s_{jj}a_{21}^2, \tag{12}$$
$$2s_{ij}a_{12}a_{11} < s_{jj}(\Delta + a_{11}^2) + s_{ii}a_{12}^2. \tag{13}$$

Note that the right hand sides of these inequalities are always positive.

1.2 A general multi-compartment model

Now suppose there are n compartments, and the numbers of cells in each compartment are denoted as $i^1, \ldots, i^n$. The transition rates $Q_k(i^1, \ldots, i^n)$ define the rates of change by a vector-valued increment $(\Delta_k i^1, \ldots, \Delta_k i^n)$.

The equilibrium and the deterministic description for the multi-compartment model

The equilibrium $(i_*^1, \ldots, i_*^n)$ is defined by n equations:

$$\sum_{k=1}^{K} Q_k(i_*^1, \ldots, i_*^n)\Delta_k i^1 = 0, \ldots, \sum_{k=1}^{K} Q_k(i_*^1, \ldots, i_*^n)\Delta_k i^n = 0. \tag{14}$$

The deterministic equations describing this system are given by

$$\dot{x}^m = \sum_{k=1}^{K} Q_k(x^1, \ldots x^n)\Delta_k i^m, \quad 1 \leq m \leq n,$$

where $x^m = \langle i^m \rangle$ for $1 \leq m \leq n$. This should be compared with the two-compartment case (2-3). The Jacobian corresponding to the equilibrium point is given by

$$J = \{a_{mj}\}, \quad a_{mj} = \sum_{k=1}^{K} \frac{\partial Q_k}{\partial x^j}\Delta_k i^m, \quad 1 \leq m, j \leq n. \tag{15}$$

Let us assume that the deterministic system under consideration is stable, and therefore the real parts of all the eigenvalues of J are negative. Next, we examine the stability conditions arising from the stochastic description.

1.2.1 Stochastic analysis of the multi-compartment model

The Kolmogorov forward equation for a multi-compartment system is a direct generalization of equation (6):

$$\begin{aligned}\dot{\varphi}_{i^1,\ldots,i^n} \quad &= \quad \sum_{k=1}^{K} \varphi_{i^1-\Delta_k i^1,\ldots,i^n-\Delta_k i^n}(t) Q_k(i^1 - \Delta_k i^1, \ldots, i^n - \Delta_k i^n) \\ &\quad -\varphi_{i^1,\ldots,i^n}(t) \sum_{k=1}^{K} Q_k(i^1, \ldots, i^n) \end{aligned} \tag{16}$$

Let us adopt the following notations for the first moments:

$$y_m = \sum_{i^1,\dots,i^n} \varphi_{i^1,\dots,i^n} i^m,$$

and for the second moments:

$$y_{qp} = \sum_{i^1,\dots,i^n} \varphi_{i^1,\dots,i^n} i^q i^p.$$

By multiplying both sides of Kolmogorov forward equation (16) by i^m and by $i^p i^q$, and summing over these indices, we obtain at the equilibrium:

$$\begin{aligned} &\sum_{k=1}^{K} \sum_{i^1,\dots,i^n} \varphi_{i^1,\dots,i^n}(t) Q_k(i^1,\dots,i^n)(i_m + \Delta_k i^m) \\ - &\sum_{k=1}^{K} \sum_{i^1,\dots,i^n} \varphi_{i^1,\dots,i^n}(t) Q_k(i^1,\dots,i^n) i^m = 0, \\ &\sum_{k=1}^{K} \sum_{i^1,\dots,i^n} \varphi_{i^1,\dots,i^n}(t) Q_k(i^1,\dots,i^n)(i_p + \Delta_k i^p)(i_q + \Delta_k i^q) \\ - &\sum_{k=1}^{K} \sum_{i^1,\dots,i^n} \varphi_{i^1,\dots,i^n}(t) Q_k(i^1,\dots,i^n) i^p i^q = 0, \end{aligned} \tag{17}$$

where $m = 1, 2, \dots, n$ and $p, q = 1, 2, \dots, n$.

Similar to the development for the two-compartment system, we assume a weak dependence of the probability rates on the cell numbers, and expand functions Q_k in Taylor series near the equilibrium. We only need to keep the terms up to the first order:

$$Q_k(i^1, i^2, \dots, i^n) \approx Q_{k*} + \sum_{j=1}^{n} \frac{\partial Q_k}{\partial x^j} \Delta_k i^j. \tag{18}$$

By following the steps of the linear noise approximation [3] (or the procedure explained in [4]), we obtain equations for the first and second moments of the cell numbers:

$$a_{m1} y_1 + a_{m2} y_2 + \dots + a_{mn} y_n = 0, \quad 1 \le m \le n, \tag{19}$$

$$\sum_{j=1}^{n} a_{pj} y_{jq} + \sum_{j=1}^{n} a_{qj} y_{pj} = -s_{pq}, \quad 1 \le p, q \le n, \tag{20}$$

where

$$a_{mj} = \sum_{k=1}^{K} \frac{\partial Q_k}{\partial x^j} \Delta_k i^m, \quad s_{pq} = \sum_{k=1}^{K} Q_{k*} \Delta_k i^p \Delta_k i^q. \tag{21}$$

There are n equations for the first moments, n equations for the 2nd moments of type y_{qq}, and $n(n-1)$ equations for mixed second order moments, $y_{qp} = y_{pq}$ with $q \neq p$ (in order to use the matrix formalism, we treat variables y_{pq} and y_{qp} as separate; the solutions for them are the same, as follows from symmetries). Let us form the vector

$$\mathbf{y} = (y_1, \dots, y_n, y_{11}, y_{12}, \dots, y_{nn})^T.$$

The equations for the moments can then be written in the form

$$\dot{\mathbf{y}} = A\mathbf{y} + F. \tag{22}$$

By the right hand side of equations (19, 20), F has a concise form:

$$F = (0, 0, \dots, 0, s_{11}, s_{12}, \dots, s_{nn})^T,$$

where the first n terms of F are 0, and the rest of the terms are elements of the matrix $[s_{ij}]_{n\times n}$ with the elements defined in (21).

The matrix A in equation (22) has the form

$$A = \begin{pmatrix} J & 0 \\ * & W \end{pmatrix},$$

where 0 denotes the matrix of zeros of dimensionality $n \times n^2$, and $*$ denotes a matrix of dimensionality $n^2 \times n$. The $n^2 \times n^2$ matrix W defines the long-term behavior of the second moments. Because of formula (20), it can be represented as the Kronecker sum

$$W = J \oplus J \equiv J \otimes I + I \otimes J, \tag{23}$$

where matrix I represents the identity matrix. Recall that the tensor product of two matrices $[A_{ij}]_{m\times p}$ and $[B_{ij}]_{n\times q}$ is a $mp \times nq$ matrix, which is defined by:

$$A \otimes B = \begin{pmatrix} a_{11}B & a_{12}B & \cdots & a_{1n}B \\ a_{21}B & a_{22}B & \cdots & a_{2n}B \\ \cdots & \cdots & \cdots & \cdots \\ a_{m1}B & a_{n2}B & \cdots & a_{mn}B \end{pmatrix}$$

First of all, we observe that similarly to the two-compartment model, the equations for the first moments completely decouple from those for the second moments, and thus the value in block $*$ of matrix A are unimportant.

Second, we note that matrix A has the same n eigenvalues as the matrix J (coming from block J in A), plus n^2 other eigenvalues defined by the matrix W. These eigenvalues are given by the sums of all pairs of the eigenvalues of matrix J. The proof of these facts can be found in [5], Theorem 4.4.5. Therefore, if all the eigenvalues of J have negative real parts, then all the eigenvalues of A also have negative real parts. Similarly, if J has eigenvalues with a nonnegative real part, then so will A. This shows that like in the two-compartment model, in the n-compartment model the stability analysis of the stochastic system gives the same results as the stability analysis of the simple deterministic system.

Finally, based on equation (22) and the form of F, we can find expressions for the second moments of the cell populations, y_{pq}. To do this, we need to solve the linear system

$$W\vec{y} = -\vec{s}, \tag{24}$$

where $\vec{y} = (y_{11}, y_{12}, \ldots, y_{nn})^T$ and $\vec{s}$ is an $n^2 \times 1$ vector with elements

$$s_{pq} = \sum_{k=1}^{K} Q_{k*} \Delta_k i^p \Delta_k i^q, \quad p, q = 1, 2, \ldots, n. \tag{25}$$

Quantities $y_{11}, \ldots, y_{nn}$ then correspond to second central moments, or the variances, of the cell populations.

2 Examples

2.1 A two-compartment model

Let us apply the methodology developed for two-compartmental systems to the example studied previously in [1], see system (1) of the main text. In this example, we assume that at each time step with probability $Q(i,j)$ one stem cell divides and with probability $1 - Q(i,j)$ one daughter cell dies. Furthermore, we assume that a stem cell can divide into two daughter cells with probability $P(i,j)$ (differentiation), and into two stem cells with probability $1 - P(i,j)$ (proliferation). We further assume a specific form of the

probabilities,

$$Q(i,j) = \frac{b}{1+h(i+j)}, \quad P(i,j) = \frac{r}{1+gj}.$$

This model has 3 transition probabilities:

$$\begin{aligned}
Q_1 &: \Delta_1 i = 1, \quad \Delta_1 j = 0, \\
&\quad Q_1(i,j) = \frac{b}{1+h(i+j)} * (1 - \frac{r}{1+gj}) = \frac{1}{4} - \frac{h}{8b} i + (-\frac{h}{8b} + \frac{g}{8r}) j \\
Q_2 &: \Delta_2 i = -1, \quad \Delta_2 j = 2, \\
&\quad Q_2(i,j) = \frac{b}{1+h(i+j)} * \frac{r}{1+gj} = \frac{1}{4} - \frac{h}{8b} i + (-\frac{h}{8b} - \frac{g}{8r}) j \\
Q_3 &: \Delta_3 i = 0, \quad \Delta_3 j = -1, \\
&\quad Q_3(i,j) = 1 - \frac{b}{1+h(i+j)} = \frac{1}{2} + \frac{h}{4b} i + \frac{h}{4b} j
\end{aligned}$$

After some calculations, we obtain the following coefficients:

$$\begin{aligned}
a_{11} &= \sum_{k=1}^{3} \frac{\partial Q_k}{\partial x} \Delta_k i = 0, \quad a_{12} = \sum_{k=1}^{3} \frac{\partial Q_k}{\partial y} \Delta_k i = \frac{g}{4r}, \\
a_{21} &= \sum_{k=1}^{3} \frac{\partial Q_k}{\partial x} \Delta_k j = -\frac{h}{2b}, \quad a_{22} = \sum_{k=1}^{3} \frac{\partial Q_k}{\partial y} \Delta_k j = -\frac{h}{2b} - \frac{g}{4r}, \\
B &= -a_{11} - a_{22} = \frac{h}{2b} + \frac{g}{4r}, \quad \Delta = a_{11}a_{22} - a_{12}a_{21} = \frac{hg}{8br}, \\
s_{ii} &= \frac{1}{2}, \quad s_{ij} = -\frac{1}{2}, \quad s_{jj} = \frac{3}{2}.
\end{aligned}$$

By formula (11) of the main text, we have

$$Var[j] = \frac{1}{2B\Delta}(s_{jj}\Delta + s_{jj}a_{11}^2 + 2s_{ij}a_{21}a_{11} + s_{ii}a_{21}^2) = \frac{r(3bg+2hr)}{g(bg+2hr)},$$

which coincides with the result of [1]. Similarly, we can prove that both methods also have the same answer for $Var[i]$.

2.2 A 3-compartment system with negative control of divisions and differentiation

Next, we provide examples of the general theory for multi-compartmental lineages. As the first example, we consider a system described in [1]. This

system consists of three compartments, which we will call stem cells, transit amplifying (TA, or "intermediate") cells, and terminally differentiated cells. Stem cells and intermediate cells are capable of symmetric divisions (either differentiations or proliferations), and differentiated cells can die at a certain rate. Therefore, there are altogether 5 processes that can happen:

$$\begin{array}{rrlll} Q_1: & \text{Differentiation of stem cells}, & \Delta_1 i^1 = -1, & \Delta_1 i^2 = 2, & \Delta_1 i^3 = 0, \\ Q_2: & \text{Proliferation of stem cells}, & \Delta_2 i^1 = 1, & \Delta_2 i^2 = 0, & \Delta_2 i^3 = 0, \\ Q_3: & \text{Differentiation of int. cells}, & \Delta_3 i^1 = 0, & \Delta_3 i^2 = -1, & \Delta_3 i^3 = 2, \\ Q_4: & \text{Proliferation of int. cells}, & \Delta_4 i^1 = 0, & \Delta_4 i^2 = 0, & \Delta_4 i^3 = 1, \\ Q_5: & \text{Death of differentiated cells}, & \Delta_5 i^1 = 0, & \Delta_5 i^2 = 0, & \Delta_5 i^3 = -1. \end{array} \quad (26)$$

The probabilities of the different events are in general functions of the variables (i^1, i^2, i^3). We say that process k is controlled by population m if function Q_k depends in non-trivial way on the variable i^m, that is,

$$\frac{\partial Q_k}{\partial i^m} \neq 0.$$

It has been shown in [1] that for stability in a three-compartment system, it is necessary to have at least three such control loops, and all three populations must be involved in the control. There are exactly 20 systems with "minimal control" (that is, only 3 control loops). Here we concentrate on one of them, namely, the system depicted in the first schematic of figure 5 in [1]. This particular system contains three negative feedback loops:

- The probability of stem cell divisions is negatively controlled by the number of stem cells.
- The probability of stem cell differentiation (vs proliferation) is negatively controlled by the intermediate cells;
- The probability of intermediate cell divisions is negatively controlled by the number of differentiated cells.

The rest of the probabilities are characterized by constant (independent of the current population) numbers.

Negative control of cell divisions has been documented in the literature. For example, in [9] it is stated that in adult neurogenesis, neural stem cell divisions are orchestrated by the mature nervous system environment, and

adult-generated neurons and glia appear to be produced on demand, rather than on a fixed schedule. In colon, a negative feedback loop via Lrig1 helps to fine-tune population size and proliferative activity of intestinal progenitor cells [10]. In [11], it is suggested that a negative control loop exists between the active stem cells and quiescent stem cells, which controls divisions of stem cells in hair, intestine, and bone marrow. These mechanisms are described in a "crowd-control" model proposed in [12].

It has been further observed that the type of cell divisions, like the division rate, is also under regulation of various types of control loops. In the context of the adult neurogenesis, it has been proposed that once generated, neural stem cell descendants can trigger some type of feedback mechanism to stop stem cell differentiation [13]. Among the candidate mechanisms, Notch signaling [14] and Prox1 expression [15] have been proposed. In [16] it is reported that haematopoietic stem cells are regulated by their mature progeny. A feedback loop is described in which platelet numbers, through regulation of available thrombopoietin levels, regulates the entry of haematopoietic stem cells into cycle.

In order to create a numerical example consistent with this system, we will choose some specific (hypothetical) functional forms of the control loops, which correspond to the regulation network described above, both in terms of topology and signs of controls. Denoting for convenience the number of stem, transit amplifying, and differentiated cells as x, y and z, we postulate the following dependencies:

$$L_1 = e^{-m_1 \epsilon x}, \quad P_1 = e^{-m_2 \epsilon y}, \quad L_2 = e^{-m_3 \epsilon z},$$

ϵ is a small parameter, quantities m_1, m_2, m_3 regulate the rate of dependence on the cell numbers, and functions L_i and P_i are the rate of division and the probability of differentiation for compartment i, respectively. Assuming that $P_2 = \alpha$, and the death rate of differentiated cells is β, both constant, we have the following functional forms for our five processes:

$$\begin{aligned} Q_1 &= L_1 P_1 = e^{-\epsilon_1 (m_1 x + m_2 y)}, \\ Q_2 &= L_1(1 - P_1) = e^{-\epsilon m_1 x}(1 - e^{-\epsilon m_2 y}), \\ Q_3 &= L_2 P_2 = e^{-\epsilon m_3 z}\alpha, \\ Q_4 &= L_2(1 - P_2) = e^{-\epsilon m_3 z}(1 - \alpha), \\ Q_5 &= \beta. \end{aligned} \tag{27}$$

The equilibrium point in this simple system can be found analytically and is given by

$$x = -\frac{1}{\epsilon m_1} \ln\left[\left(\alpha - \frac{1}{2}\right)\frac{\beta}{\alpha}\right], \quad y = \frac{\ln 2}{\epsilon m_2}, \quad z = -\frac{1}{\epsilon m_3} \ln\left[\frac{\beta}{2\alpha}\right]. \tag{28}$$

It is positive as long as $\alpha > 1/2$. Figure 1(a) shows a typical numerical run of this model for a particular parameter set. The three populations, the stem cells, the TA cells, and the differentiated cells, are plotted as a function of the time-step of the simulation. The variance in the three cell populations can be obtained either as a solution of system (24) of the main text or by formula (26) of the main text, and can be obtained as a (slightly long) closed form expression, which we omit here.

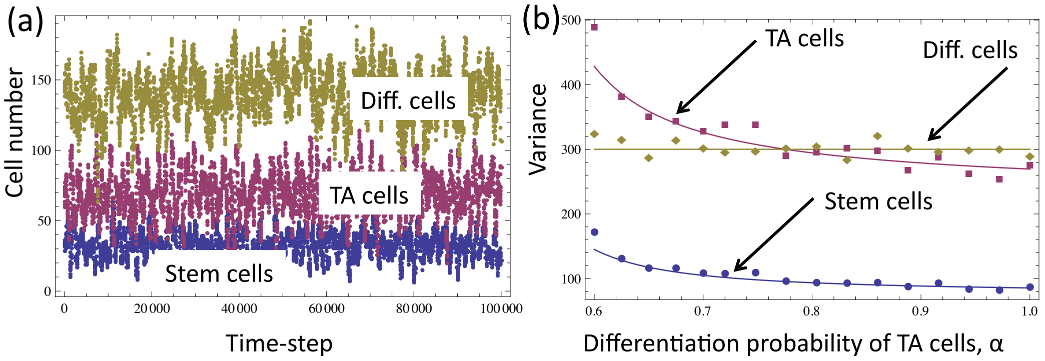

Figure 1: Numerical and analytical studies of a three-compartment stem cell system, (27). (a) A typical numerical simulation; the three populations, the stem cells, the TA cells, and the differentiated cells, are plotted as a function of the time-step of the simulation. The initial condition is given by the theoretically calculated means, equations (28). The parameters are $\alpha = 0.95$, $\beta = 0.95$, $\epsilon = 0.005$, $m_1 = 5, m_2 = 2, m_3 = 1$. (b) The variance of the three population sizes, as a function of parameter α. The solid lines are the theoretical curves, and the points are calculated numerically, from at least 10^5 time-steps. The rest of the parameters are as in part (a).

There are several points that follow from this analysis. The system is only stable if the transit amplifying cells differentiate more than 50% of the time. As the controls become weaker (ϵ decreases), the means increase

as $1/\epsilon$, and the standard deviations as $1/\sqrt{\epsilon}$. That is, as controls become weaker, the relative size of the fluctuations decreases. The probability of cell death, β, does not influence the variances, but only the means of stem and differentiated cells. The mean number of transit amplifying cells and the variance of the differentiated cells do not depend on the probability of TA differentiation, α. The variance of the stem and transit amplifying cells decays with the probability of differentiation, α. The latter trends are seen in figure 1(b), where we plot the variance of the three cell populations as a function of α. The solid lines correspond to the analytical solution, and the dots show the numerically calculated values of the variance.

2.3 A 3-compartment system with positive control of differentiation and negative control of divisions

Our second example again comes from one of the minimal (3-compartment, 3-control loop) systems of [1] and includes the five processes listed in (37). We consider the tenth schematic of figure 5 in [1], which contains two positive and one negative feedback loops:

- The probability of stem cell differentiation is positively controlled by the number of stem cells.
- The probability of intermediate cell differentiation is positively controlled by the number of intermediate cells;
- The probability of stem cell divisions is negatively controlled by the number of differentiated cells.

The rest of the probabilities are characterized by constant (independent of the current population) numbers.

Positive control of differentiation by cell numbers has been discussed in the literature. Extracellular signals and the micro-environment constitute a niche, in which stem cells compete for limiting concentrations of growth factors, thereby maintaining a balance between self-renewal and differentiation. Wnt protein has been shown to promote stem cell self-renewal [17]. If such self-renewal-promoting factor is secreted by the cells of the stem cell niche, then the stem cells in the proximity of the source of the signal will tend to self-renew, while stem cells further away will tend to differentiate. This corresponds to a positive control of differentiation by the stem cells: the

more stem cells there are, the more likely it is that a given stem cell will find itself relatively far from the niche, and thus its probability to differentiate will increase. Furthermore, in some cases, mechanical strain has been shown to increase cell differentiation [18, 19, 20], leading to the same trend. Finally, it has been suggested that stem cells have to be spatially localized to their niches, which keeps them protected from the differentiating influences of the surrounding microenvironment [21]. Therefore, as the number of stem cells increases, the probability to be exposed to the differentiation signals from the outside increases, resulting in a positive control loop.

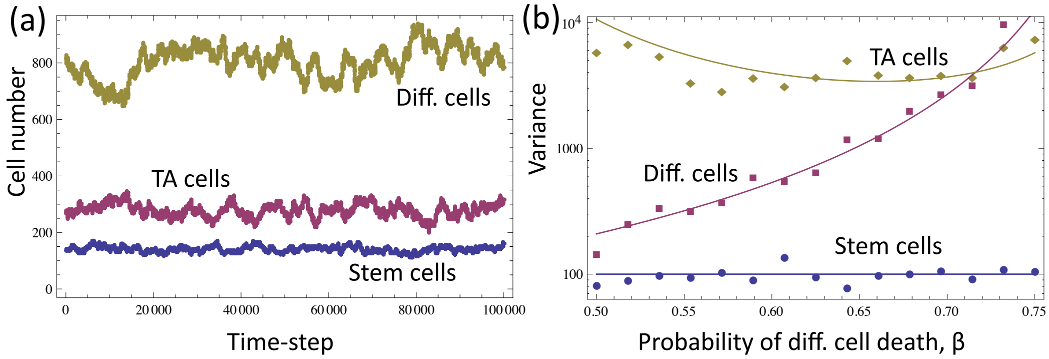

Figure 2: Numerical and analytical studies of a three-compartment stem cell system, (29). (a) A typical numerical simulation; the three populations, the stem cells, the TA cells, and the differentiated cells, are plotted as a function of the time-step of the simulation. The initial condition is given by the theoretically calculated means, equations (30). The parameters are $\gamma = 0.4$, $\beta = 0.6$, $\epsilon = 0.005$. (b) The variance of the three population sizes, as a function of parameter β. The solid lines are the theoretical curves (formula (26) of the main text), and the points are calculated numerically, from at least 10^5 time-steps. The rest of the parameters are as in part (a).

Now we return to the example at hand. To be specific, we postulate the following dependencies:

$$L_1 = \frac{1}{1+\epsilon z}, \quad P_1 = 1 - e^{-\epsilon x}, \quad P_2 = 1 - e^{-\epsilon y}.$$

Assuming that $L_2 = \gamma$, and the death rate of differentiated cells is β, both constant, we have the following functional forms for our five processes:

$$\begin{aligned}
Q_1 &= L_1 P_1 = \frac{1}{1+\epsilon z}(1 - e^{-\epsilon x}), \\
Q_2 &= L_1(1 - P_1) = \frac{1}{1+\epsilon z} e^{-\epsilon x}, \\
Q_3 &= L_2 P_2 = \gamma(1 - e^{-\epsilon y})\alpha, \qquad (29) \\
Q_4 &= L_2(1 - P_2) = \gamma e^{-\epsilon y}, \\
Q_5 &= \beta,
\end{aligned}$$

The equilibrium point in this simple system can be found analytically and is given by

$$x = -\frac{\ln 2}{\epsilon}, \quad y = -\frac{1}{\epsilon}\left[1 - \frac{\beta}{2\gamma}\right], \quad z = \frac{1+\gamma-\beta}{\epsilon(\beta-\gamma)}. \qquad (30)$$

It is positive as long as $\gamma < \beta < 2\gamma$. Figure 1(a) shows a typical numerical run of this model for a particular parameter set. Part (b) of the figure shows the variance of the three populations as a function of parameter β, the differentiated cell death rate. Again, the variance can be obtained either as a solution of system (24) or by formula (26) of the main text; for this example the analytical solution is slightly cumbersome and is not presented explicitly.

3 Stability and variance calculations

In this paper we apply variance calculations of different cell populations to compare different parameters and networks with each other. A question arises on the similarities and difference between this approach and the usual stability analysis of the deterministic system. Using two simple examples, we will demonstrate that the two methods are compatible, but the analysis of variances can provide further insights into cell population behavior.

We will consider the two minimal control systems described in [1]. There are two populations, stem cells (x) and differentiated cells (y). The division rate of stem cells is denoted by $L(x, y)$, the differentiation probability $P(x, y)$, and death rate as $D(x, y)$. The deterministic system is given by

$$\dot{x} = L(1 - 2P), \qquad (31)$$

$$\dot{y} = 2LP - D. \qquad (32)$$

The four controls that enter the calculations of stability and variances are

$$q_x = \frac{\partial(L-D)}{\partial x}, \quad q_y = \frac{\partial(L-D)}{\partial y}, \quad p_x = \frac{\partial P}{\partial x}, \quad p_x = \frac{\partial P}{\partial y},$$

see [1] for explicit expressions for the variances. Each of the two minimal systems only contains two nonzero controls.

The first minimal control corresponds to $q_x = 0, p_y = 0, p_x > 0, q_y < 0$. In this case (see figure 3), the eigenvalues of the linear system are minimized for p_x large and $|q_y|$ large (and q_y negative), see panels (a) and (b) of figure 3 (respectively). Simultaneous minimization of both eigenvalues (which corresponds to increased stability) happens as one increases p_x and decreases negative q_y.

When we consider the variances of stem cells (panel (c)) and differentiated cells (panel (d)), we can see that their simultaneous minimizations also occurs for large p_x and large negative q_y. Therefore, both types of analysis point to the same optimization procedure for the parameters, which is not surprising. Both types of analysis suggest that increasing the absolute values of quantities p_x and q_y will lead to an increased overall stability and decreases variance in the system.

In this case, the only advantage of the variance method is that it allows us to calculate the numerical values of the variances. If biological information is available on the acceptable % change of a population size in a given tissue, this could be directly translated into constraints on the system parameters, given the analytic expressions for the variances.

The second minimal control corresponds to $p_x = 0, q_y = 0, p_y < 0, q_x < 0$. In this case (see figure 4), we have a different pictures. One of the eigenvalues (panel (a)) is minimized when both q_x and p_y are large and negative. The other eigenvalue (panel (b)) is minimized when q_x is small and negative and p_y is large and negative.

The same pictures is observed when we consider the two variances (panels 4(c) and (d)). Again, this is not surprising, and we expect to obtain a qualitatively similar result when using both methods. In the present case however the knowledge of the variances allows us to formulate meaningful minimization problems that have biologically relevant solutions. For example, the quantity $\sqrt{Var(x)}/E(x) + \sqrt{Var(y)}/E(y)$ is the sum of two relative

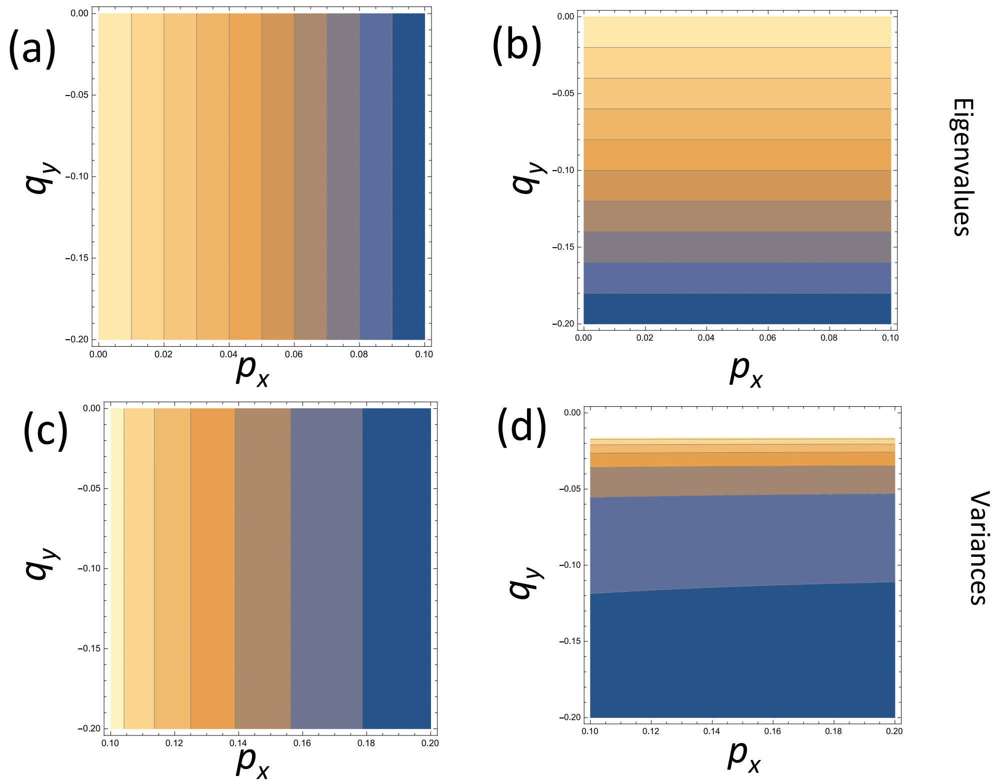

Figure 3: Stability and variance analysis of the first minimal system. Heat plots of the two linear eigenvalues ((a) and (b)) and variances of the stem cell population (c) and differentiated cell population (d) are presented, depending on the controls p_x and q_y. Blue corresponds to lower values.

standard deviations of the two cell populations. Figure 5 shows that this quantity is minimized when both p_x and q_y have the largest magnitude.

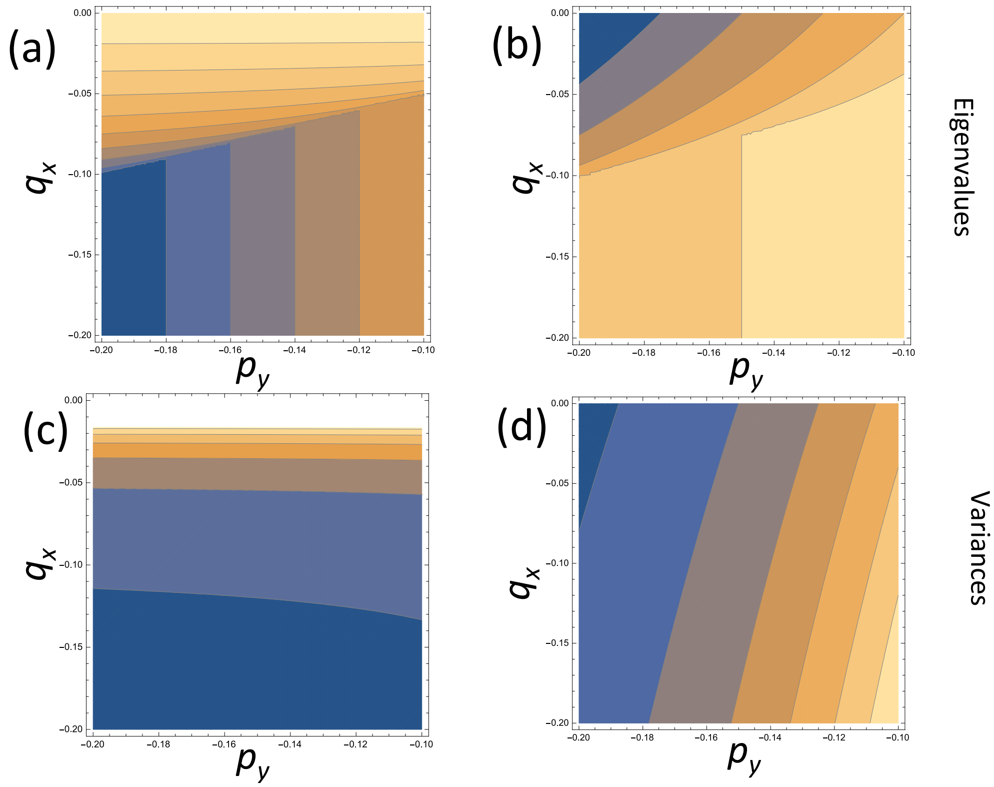

Figure 4: Stability and variance analysis of the second minimal system. Heat plots of the two linear eigenvalues ((a) and (b)) and variances of the stem cell population (c) and differentiated cell population (d) are presented, depending on the controls p_y and q_x. Blue corresponds to lower values.

4 Mouse airway epithelium: examples of networks, their stability, and recovery dynamics

Let us study the dynamics of the airway epithelium by including all the processes listed in Table 1 of the main text. System (14) of the main text can be solved to express three of the equilibrium values of the control parameters

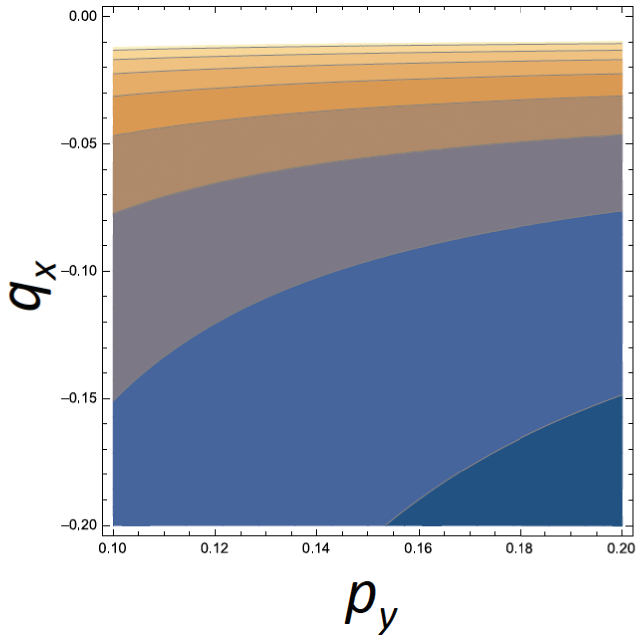

Figure 5: Heat plot of the quantity $\sqrt{Var(x)}/E(x) + \sqrt{Var(y)}/E(y)$ for the second minimal system. Blue corresponds to lower values.

in terms of the other 8. Solving for $Q_{4,0}, Q_{5,0}, Q_{6,0}$, we obtain

$$
\begin{aligned}
2Q_{4,0} &= -2Q_{2,0} + Q_{8,0} - Q_{10,0} - Q_{11,0}, && (33)\\
2Q_{5,0} & \quad -2Q_{1,0} - 2Q_{3,0} + 2Q_{7,0} + Q_{8,0} - 2Q_{9,0} - Q_{10,0} - Q_{11,0}, && (34)\\
Q_{6,0} &= Q_{1,0} + Q_{2,0} - Q_{3,0}. && (35)
\end{aligned}
$$

In the most general case we can assume that all the processes are controlled by all the variables, that is, all derivatives Q_{ix}, Q_{iy}, Q_{iz} are nonzero for $1 \leq i \leq 11$.

Stability of the solution is determined by calculating the eigenvalues of matrix J defined by equation (15) of the main text, which in this most general case, is equivalent to solving a cubic equation. Stability, which is equivalent to negativity of the real part of all the eigenvalues, can be tested by using the Routh Hurwitz criterion.

A simplification is obtained if we assume that CilCs do not exert control over any processes except for their own death, that is, $Q_{iz} = 0$ for $i \neq 8$. In this case, matrix J is separable, and one of the eigenvalues has a simple

form, $\lambda_3 = -Q_{8z}$. In this case, the assumption $Q_{8z} > 0$ is the required for stability. The other two eigenvalues have a negative real part if

$$\begin{aligned}
& Q_{1x} + Q_{2x} - Q_{3x} - Q_{6x} - 2Q_{1y} + Q_{4y} - Q_{5y} + Q_{6y} + Q_{7y} - Q_{9y} > 0, \\
- \quad & Q_{4x}Q_{1y} + Q_{5x}Q_{1y} + Q_{6x}Q_{1y} - Q_{7x}Q_{1y} + Q_{9x}Q_{1y} - Q_{4x}Q_{2y} + Q_{5x}Q_{2y} \\
- \quad & Q_{6x}Q_{2y} - Q_{7x}Q_{2y} + Q_{9x}Q_{2y} + Q_{4x}Q_{3y} - Q_{5x}Q_{3y} + Q_{6x}Q_{3y} + Q_{7x}Q_{3y} \\
- \quad & Q_{9x}Q_{3y} - Q_{6x}Q_{4y} + Q_{6x}Q_{5y} + Q_{4x}Q_{6y} - Q_{5x}Q_{6y} + Q_{7x}Q_{6y} - Q_{9x}Q_{6y} \\
- \quad & Q_{6x}Q_{7y} + Q_{1x}(2Q_{2y} - 2Q_{3y} + Q_{4y} - Q_{5y} - Q_{6y} + Q_{7y} - Q_{9y}) \\
+ \quad & Q_{2x}(-2Q_{1y} + Q_{4y} - Q_{5y} + Q_{6y} + Q_{7y} - Q_{9y}) + Q_{6x}Q_{9y} \\
+ \quad & Q_{3x}(2Q_{1y} - Q_{4y} + Q_{5y} - Q_{6y} - Q_{7y} + Q_{9y}) > 0. \qquad (36)
\end{aligned}$$

Next, let us restrict ourselves to a simpler case where only a subset of x- and y- controls are nonzero, assuming the following possibly negative controls:

$$Q_{1y}, Q_{2y}, Q_{3y}, Q_{4x}, Q_{5x}, Q_{6x}, Q_{9y}, Q_{10,y}, Q_{11,y} \leq 0, \qquad (37)$$

together with the condition

$$Q_{8z} > 0, \qquad (38)$$

as follows from the previous analysis. We assume that the rest of the controls are zero. The stability conditions simplify to

$$\begin{aligned}
Q_{8z} &> 0, \qquad (39) \\
Q_{6x} + 2Q_{1y} + Q_{9y} &< 0, \qquad (40) \\
-Q_{4x}(Q_{1y} + Q_{2y} - Q_{3y}) + Q_{5x}(Q_{1y} + Q_{2y} - Q_{3y}) & \\
+Q_{6x}(Q_{1y} - Q_{2y} + Q_{3y} + Q_{9y}) &> 0. \qquad (41)
\end{aligned}$$

While the first two of these conditions are always satisfied as long as the controls have a correct sign, the third condition is more restrictive.

4.1 Only symmetric divisions

Let us assume that only symmetric divisions take place for both SCs and SecrCs, see figure 6. This means that processes Q_9, Q_{10}, Q_{11} do not take place, and at homeostasis we have

$$\begin{aligned}
2Q_{4,0} &= -2Q_{2,0} + Q_{8,0}, \qquad (42) \\
2Q_{5,0} & \quad -2Q_{1,0} - 2Q_{3,0} + 2Q_{7,0} + Q_{8,0}, \qquad (43) \\
Q_{6,0} &= Q_{1,0} + Q_{2,0} - Q_{3,0}. \qquad (44)
\end{aligned}$$

The stability condition (under the assumption on the signs as in (37,38)) is now given by

$$-Q_{4x}(Q_{1y}+Q_{2y}-Q_{3y})+Q_{5x}(Q_{1y}+Q_{2y}-Q_{3y})+Q_{6x}(Q_{1y}-Q_{2y}+Q_{3y})>0. \quad (45)$$

The variance of the three populations can be calculated analytically by using equation (24) of the main text. The expressions are quite cumbersome and are not provided here.

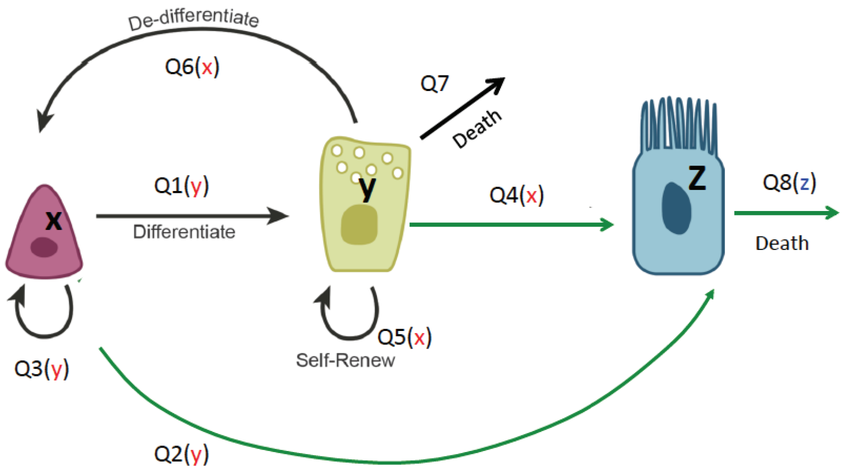

Figure 6: A schematic of cellular processes under the assumption of purely **symmetric** divisions of SCs and SecrCs. In figures 6 and 8, divisions are symmetric if denoted by solid arrows, and asymmetric if denoted by dashed arrows. Low-rate process associated with the slow dynamics of CilCs are depicted in green.

We have performed numerical simulations to investigate if such a system is capable of exhibiting homeostasis and recovery behavior consistent with observations. To generate such a system, assigned 11 parameter values: 4 values for the equlibrium rates, $Q_{1,0}, Q_{2,0}, Q_{3,0}, Q_{7,0}$ (we set $Q_{8,0}=0$); 3 negative controls by SecrCs (Q_{1y}, Q_{2y}, Q_{3y}); 3 negative controls by SCs (Q_{4x}, Q_{5x}, Q_{6x}); and the control of the death rate of CilCs, $Q_{8z}>0$. The values were assigned randomly (with the correct sign), from a uniform distribution. We also had to make sure that the death of CilCs is low (see the main text), and therefore their production (processes $Q2$ and $Q4$) are also

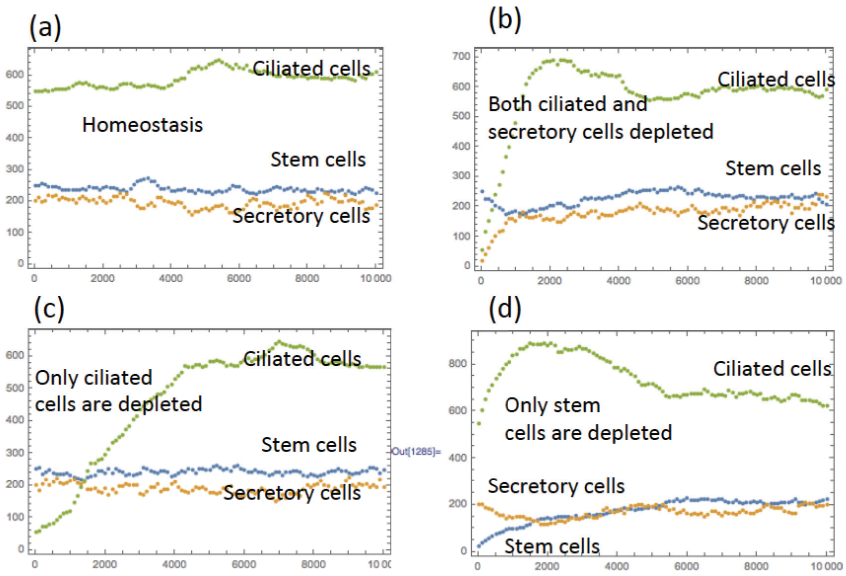

Figure 7: Homeostasis and recovery dynamics under the assumption of purely **symmetric** divisions. Typical runs with different initial conditions are presented. (a) Homeostasis: the initial condition is given by $x = x_0, y = y_0, z = z_0$. (b) Experimental scenario I: the initial condition is given by $x = x_0, y = y_0, z = 0.1\, z_0$. (c) Scenario II: $x = 0.1\, x_0, y = y_0, z = z_0$. (d) Scenario III: $x = x_0, y = 0.1\, y_0, z = 0.1\, z_0$. Parameters are generated randomly by means of the procedure described in the text.

slow; we achieved that by multiplying the corresponding coefficients by 10^{-3}. The affected rates are marked by green arrows in figure 6. Stability condition (45) is easy to satisfy; about 70% of all randomly generated systems satisfy it, so the system is relatively robust.

A typical run in homeostasis for such a system is presented in figure 7(a). Further, examples of recovery dynamics according to scenarios (I), (II), and (III) are shown in figures 7(b-d), see the main text for the details of the different scenarios and corresponding experimental findings. The conclusion is that a system with purely symmetric divisions can be stable, and is capable of reproducing qualitatively correct recovery behavior under three different types of injury.

4.2 Only asymmetric divisions

Let us now assume that only asymmetric divisions take place for both SCs and SecrCs, see figure 8. This means that processes Q_1, Q_2, Q_3, Q_4, Q_5 (symmetric divisions) do not take place, and instead we have processes Q_9, Q_{10}, Q_{11} (asymmetric divisions). At homeostasis, the solution must satisfy

$$Q_{6,0} = 0, \quad Q_{9,0} = Q_{7,0}, \quad Q_{11,0} = Q_{8,0} - Q_{10,0}.$$

The general stability conditions simplify to

$$Q_{6x} < 0, \quad Q_{9y} < 0, \quad Q_{8z} > 0,$$

which are satisfied as long as the controls have correct signs, as defined by (37,38); no additional stability conditions are required.

The expressions for the variance obtained from equation (24) of the main text are as follows:

$$\begin{aligned} y_{11} &= 0, \\ y_{22} &= -\frac{Q_{7,0}}{Q_{9y}}, \\ y_{33} &= \frac{Q_{7,0}Q_{10y}^2 + Q_{8,0}Q_{9y}(Q_{9y} - Q_{8z})}{Q_{9y}Q_{8z}(Q_{9y} - Q_{8z})}. \end{aligned}$$

As expected, the variance of SCs, y_{11}, is zero because SCs only divide asymmetrically. The variance of SecrCs, y_{22}, is nonzero because these cells can die and de-differentiate, and get replenished by divisions of SCs. We further observe, by taking the derivatives of y_{22} and y_{33} with respect to Q_{9y}, that tighter negative control of SC divisions by the SecrC population, which corresponds to larger absolute values of Q_{9y}, leads to smaller variances of SecrC and CilC populations, that is smaller y_{22} and y_{33} values. Smaller death rates of SecrCs ($Q_{7,0}$) will decrease both variances. On the other hand, SecrC control of SC divisions producing CilCs, or controls of de-differentiation and SecrC divisions do not help decrease variance.

As with purely symmetric divisions, we have tested if the asymmetrically dividing system is capable of showing realistic homeostasis and recovery dynamics. We have performed numerical simulations by randomly generating 8 parameter values: 2 values for the equlibrium rates of processes $Q_{7,0}, Q_{10,0}$ (we again set $Q_{8,0} = 0$); 3 negative controls by SecrCs (Q_{9y}, Q_{10y}, Q_{11y}); 2

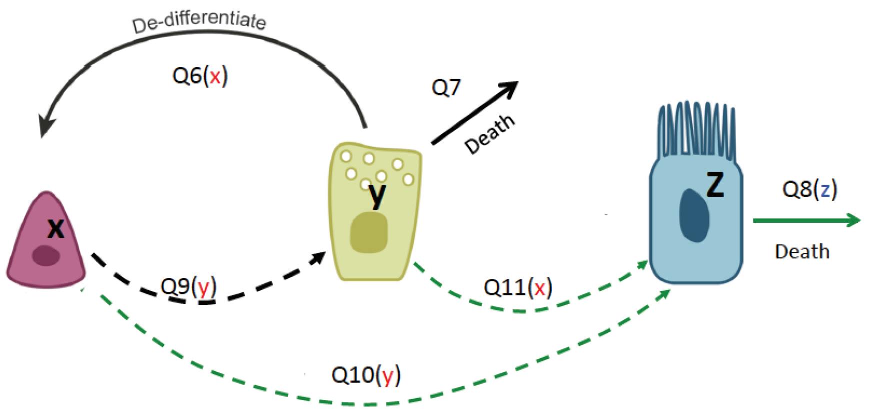

Figure 8: A schematic of cellular processes under the assumption of purely **asymmetric** divisions of SCs and SecrCs. In figures 6 and 8, divisions are symmetric if denoted by solid arrows, and asymmetric if denoted by dashed arrows. Low-rate process associated with the slow dynamics of CilCs are depicted in green.

negative controls by stem cells (Q_{11x}, Q_{6x}), and the death rate control of CilCs, $Q_{8z} > 0$. Again, to ensure slow dynamics of CilCs, we multiplied all rates associated with these cells (Q_8, Q_{10}, Q_{11}) by 10^{-3}. The affected rates are marked by green arrows in figure 8. 100% of systems generated in this way are stable.

A typical run in homeostasis for such a system is presented in figure 9(a). Further, examples of recovery dynamics according to scenarios (I), (II), and (III) are shown in figures 9(b-d), see the main text for the details of the different scenarios and corresponding experimental findings. Again, the conclusion is that a system with purely asymmetric divisions can be stable, and is capable of reproducing qualitatively correct recovery behavior under three different types of injury.

5 Minimization of variance as a function of the equilibrium rates

This section provides a theoretical foundation for the claim that asymmetric SC divisions in the airway epithelium system correspond to the minimal

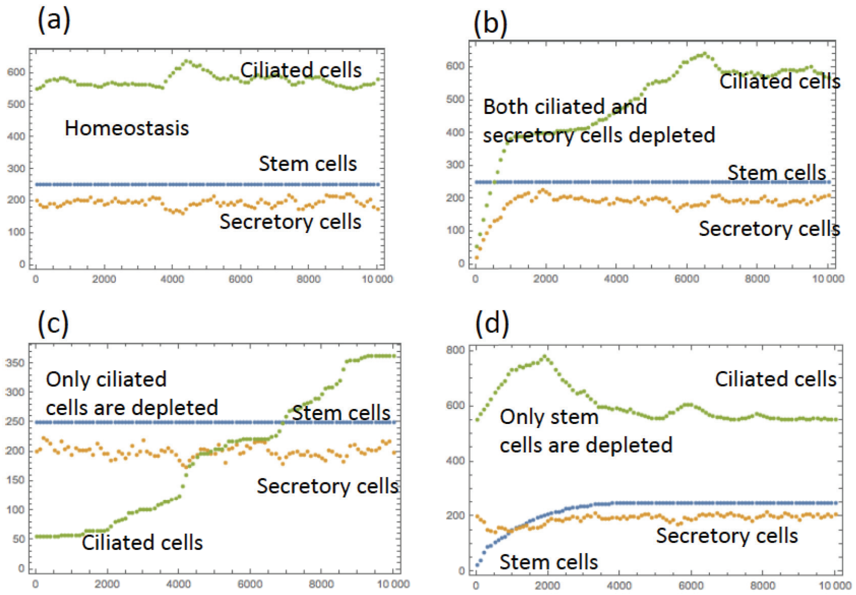

Figure 9: Homeostasis and recovery dynamics under the assumption of purely **asymmetric** divisions. Typical runs with different initial conditions are presented. (a) Homeostasis: the initial condition is given by $x = x_0, y = y_0, z = z_0$. (b) Experimental scenario I: the initial condition is given by $x = x_0, y = y_0, z = 0.1\, z_0$. (c) Scenario II: $x = 0.1\, x_0, y = y_0, z = z_0$. (d) Scenario III: $x = x_0, y = 0.1\, y_0, z = 0.1\, z_0$. Parameters are generated randomly by means of the procedure described in the text.

variance of the cell numbers. We perform the analysis in two cases: (1) First we assume that the smallness of rates Q_2, Q_4, Q_8, Q_{10} and Q_{11} implies the smallness of their derivatives at the equilibrium. (2) Then we remove this assumption and present a general proof.

5.1 Assumption of the smallness of derivatives

First, we study the stability of this effectively two-compartment system. The eigenvalues of the Jacobian (15) satisfy the equations

$$\lambda^2 + \alpha\lambda + \beta = 0,$$

where we denoted

$$\alpha = -(Q_{6x} + 2Q_{1y} + Q_{9y}) > 0, \quad (46)$$

$$\beta = Q_{5x}(Q_{1y} - Q_{3y}) + Q_{6x}(Q_{1y} + Q_{3y} + Q_{9y}). \quad (47)$$

The eigenvalues have a negative real part if $\beta > 0$. Expressions for the variances of SCs and SecrCs can be obtained by using equation (6) of the main text, they turn out to be linear functions of $Q_{1,0}$ and $Q_{3,0}$. We can write these expressions in the following form:

$$Var[x] = c_1 Q_{1,0} + c_3 Q_{3,0} + c_0, \quad (48)$$

$$Var[y] = d_1 Q_{1,0} + d_3 Q_{3,0} + d_0, \quad (49)$$

where c_1, c_3, c_0 and d_1, d_3, d_0 are independent of $Q_{1,0}$ and $Q_{3,0}$ (but depend on the derivatives of the controls). The goal is to find the values $Q_{1,0}$ and $Q_{3,0}$ that minimize he variances, but at the same time are compatible with existence and stability of solutions. For each of the variance expressions, this comprises a linear minimization problem with a finite 2D feasible region defined by the positivity of all the control magnitudes, including the expressions in (32-33) of the main text:

$$Q_{3,0} \leq A - Q_{1,0}, \quad A = Q_{7,0} - Q_{9,0} \geq 0, \quad (50)$$

$$Q_{3,0} \leq Q_{1,0}, \quad (51)$$

$$Q_{3,0} \geq 0. \quad (52)$$

In the $(Q_{1,0}, Q_{3,0})$ coordinates this is a triangular region with the corners at $(0,0)$, $(A,0)$, and $(A/2, A/2)$. The minimum of functions $Var[x]$ and $Var[y]$ can be found by evaluating the expressions (48) and (49) in the three corners, and finding the lowest value. For example, for $Var[x]$, the three values are respectively

$$c_0, \quad c_0 + Ac_1, \quad c_0 + A(c_1 + c_3)/2.$$

The minimum corresponds to corner $(0,0)$ if $c_1 > 0$ and $c_1 + c_3 > 0$. Similarly, the minimum of $Var[y]$ is achieved in the zero corner if $d_1 > 0$ and $d_1 + d_3 > 0$.

Using some algebra we find:

$$\begin{aligned} c_1 &= -\frac{2\beta + Q_{5x}(Q_{5x}+Q_{6x})}{\beta(Q_{6x}+2Q_{1y}+Q_{9y})} > 0, && (53)\\ c_1 + c_3 &= -\frac{\beta + Q_{5x}^2}{\beta(Q_{6x}+2Q_{y1}+Q_{9y})} > 0, && (54)\\ d_1 &= -\frac{\beta + (Q_{1y}+Q_{3y}+Q_{9y})(2Q_{3y}+Q_{9y})}{\beta(Q_{6x}+2Q_{1y}+Q_{9y})} > 0, && (55)\\ d_1 + d_3 &= -\frac{\beta + (Q_{1y}+Q_{3y}+Q_{9y})^2}{\beta(Q_{6x}+2Q_{1y}+Q_{9y})} > 0, && (56)\end{aligned}$$

where we used the negativity of the partial derivatives and stability condition for this system, $\beta > 0$. This means both SC and SecrC variances are minimized for zero values of $Q_{1,0}$ and $Q_{3,0}$, which in turns suggests that the optimal SC division pattern from the point of view of homeostasis maintenance is asymmetric divisions.

5.2 General proof

Now we assume that even though the equilibrium values of the rates that influence the dynamics of CilCs are small, the derivatives of these rates at the equilibrium may not be small. The analysis of the previous subsection can be carried out in the same way, but now instead of expressions (53-56) we have

$$\begin{aligned} c_1 &= -\frac{2\beta + (Q_{5x}-Q_{4x})(Q_{5x}-Q_{4x}+Q_{6x})}{\beta(Q_{6x}+2Q_{1y}+Q_{9y})}, && (57)\\ c_1 + c_3 &= -\frac{\beta + (Q_{5x}-Q_{4x})^2}{\beta(Q_{6x}+2Q_{y1}+Q_{9y})} > 0, && (58)\\ d_1 &= -\frac{\beta + (Q_{1y}+Q_{3y}-Q_{2y}+Q_{9y})(2(Q_{3y}-Q_{2y})+Q_{9y})}{\beta(Q_{6x}+2Q_{1y}+Q_{9y})}, && (59)\\ d_1 + d_3 &= -\frac{\beta + (Q_{1y}+Q_{3y}-Q_{2y}+Q_{9y})^2}{\beta(Q_{6x}+2Q_{1y}+Q_{9y})} > 0, && (60)\end{aligned}$$

where

$$\beta = -Q_{4x}(Q_{1y}+Q_{2y}-Q_{3y}) + Q_{5x}(Q_{1y}+Q_{2y}-Q_{3y}) + Q_{6x}(Q_{1y}-Q_{2y}+Q_{3y}+Q_{9y}) > 0.$$

One can see that while quantities $c_1 + c_3$ and $d_1 + d_3$ are always positive, the signs of coefficients c_1 and d_1 are harder to determine.

It turns out that the non-negativity of these quantities can be proven in general, for any stem cell system. For the proof, it is convenient to use the solution for the second moments given by equation (26) of the main text:

$$Y = \int_0^\infty \exp(Jt) S \exp(J^T t)\, dt, \tag{61}$$

It is clear that $Y = \{y_{pq}\}$, the matrix of moments, depends linearly on the equilibrium rates, Q_{k*}, through the matrix $S = \{s_{pq}\} = \{\sum_{k=1}^{K} Q_{k*}\Delta_k i^p \Delta_k i^q\}$. Let us determine the sign in front of the coefficients that multiply the equilibrium rates, Q_{l*}:

$$\frac{\partial Y}{\partial Q_{l*}} = \int_0^\infty \exp(Jt) \frac{\partial S}{\partial Q_{l*}} \exp(J^T t)\, dt, \tag{62}$$

where

$$\frac{\partial S}{\partial Q_{l*}} = \{\Delta_l i^p \Delta_l i^q\},$$

a cross-product of vector $(\Delta_l i^1, \dots, \Delta_l i^n)$ with itself. Note that, by virtue of being a cross-product, this is a positive semi-definite matrix.

Let us present the Jacobian matrix J and its transpose by means of its singular value decomposition:

$$J = UWV^T, \quad J^T = VWU^T,$$

where W is a diagonal matrix and U and V are unitary matrices. Then the expression inside the integral in (61) can be written as

$$Ue^{Wt}V^T \frac{\partial S}{\partial Q_{l*}} V E^{Wt} U^T = P \frac{\partial S}{\partial Q_{l*}} P^T,$$

where we denoted

$$P = Ue^{Wt}V^T.$$

Then we have for the diagonal entries of Y,

$$\frac{\partial y_{ii}}{\partial Q_{l*}} = \int_0^\infty (P_i) \frac{\partial S}{\partial Q_{l*}} (P_i)^T\, dt \geq 0,$$

where (P_i) denotes the ith line of matrix P. The inequality follows from the fact that matrix $\frac{\partial S}{\partial Q_{l*}}$ is positive semi-definite.

As a consequence, expressions (57) and (59) are nonnegative, and the variances are minimized by $Q_{1,0} = Q_{3,0} = 0$.

References

1. Komarova NL. Principles of regulation of self-renewing cell lineages. PloS one. 2013;8(9):e72847.

2. Lander AD, Gokoffski KK, Wan FY, Nie Q, Calof AL. Cell lineages and the logic of proliferative control. PLoS Biol. 2009 Jan;7:e15.

3. Kampen Nv. A power series expansion of the master equation. Canadian Journal of Physics. 1961;39(4):551–567.

4. Yang J, Sun Z, Komarova NL. Analysis of stochastic stem cell models with control. Mathematical biosciences. 2015;266:93–107.

5. Horn R, Johnson CR. Topics in Matrix Analysis. New York: Cambridge University Press; 1991.

6. Barnett S, Storey C. Some applications of the Lyapunov matrix equation. IMA Journal of Applied Mathematics. 1968;4(1):33–42.

7. Barnett S. Introduction to mathematical control theory. Clarendon Press; 1975.

8. Laub AJ. Matrix analysis for scientists and engineers. Philadelphia: Siam; 2005.

9. Hsieh J. Orchestrating transcriptional control of adult neurogenesis. Genes & Development. 2012;26(10):1010–1021.

10. Ordóñez-Morán P, Huelsken J. Lrig1: a new master regulator of epithelial stem cells. The EMBO Journal. 2012;.

11. Li L, Clevers H. Coexistence of quiescent and active adult stem cells in mammals. Science. 2010;327(5965):542–545.

12. Dehay C, Kennedy H. Cell-cycle control and cortical development. Nature Reviews Neuroscience. 2007;8(6):438–450.

13. Liu M, Pleasure SJ, Collins AE, Noebels JL, Naya FJ, Tsai MJ, et al. Loss of BETA2/NeuroD leads to malformation of the dentate gyrus and epilepsy. Proceedings of the National Academy of Sciences. 2000;97(2):865–870.

14. Alvarez-Buylla A, Lim DA. For the long run: maintaining germinal niches in the adult brain. Neuron. 2004;41(5):683–686.

15. Lavado A, Lagutin OV, Chow LML, Baker SJ, Oliver G. Prox1 is required for granule cell maturation and intermediate progenitor maintenance during brain neurogenesis. PLoS biology. 2010;8(8):e1000460.

16. de Graaf CA, Kauppi M, Baldwin T, Hyland CD, Metcalf D, Willson TA, et al. Regulation of hematopoietic stem cells by their mature progeny. Proceedings of the National Academy of Sciences. 2010;107(50):21689–21694.

17. Nusse R. Wnt signaling and stem cell control. Cell research. 2008;18(5):523–527.

18. Guilak F, Cohen DM, Estes BT, Gimble JM, Liedtke W, Chen CS. Control of stem cell fate by physical interactions with the extracellular matrix. Cell Stem Cell. 2009;5(1):17–26.

19. Simmons CA, Matlis S, Thornton AJ, Chen S, Wang CY, Mooney DJ. Cyclic strain enhances matrix mineralization by adult human mesenchymal stem cells via the extracellular signal-regulated kinase (ERK1/2) signaling pathway. Journal of biomechanics. 2003;36(8):1087–1096.

20. Sen B, Xie Z, Case N, Ma M, Rubin C, Rubin J. Mechanical strain inhibits adipogenesis in mesenchymal stem cells by stimulating a durable β-catenin signal. Endocrinology. 2008;149(12):6065–6075.

21. Adams G, Scadden D. A niche opportunity for stem cell therapeutics. Gene therapy. 2007;15(2):96–99.
